# What Affects the Quality of Score Transformations? Potential Issues in True-Score Equating Using the Partial Credit Model

**DOI:** 10.1177/00131644221143051

**Published:** 2023-01-13

**Authors:** Carolina Fellinghauer, Rudolf Debelak, Carolin Strobl

**Affiliations:** 1 University of Zurich, Switzerland

**Keywords:** Rasch analysis, simulation study, equating, true-score equating, multidimensionality, common person design

## Abstract

This simulation study investigated to what extent departures from construct similarity as well as differences in the difficulty and targeting of scales impact the score transformation when scales are equated by means of concurrent calibration using the partial credit model with a common person design. Practical implications of the simulation results are discussed with a focus on scale equating in health-related research settings. The study simulated data for two scales, varying the number of items and the sample sizes. The factor correlation between scales was used to operationalize construct similarity. Targeting of the scales was operationalized through increasing departure from equal difficulty and by varying the dispersion of the item and person parameters in each scale. The results show that low similarity between scales goes along with lower transformation precision. In cases with equal levels of similarity, precision improves in settings where the range of the item parameters is encompassing the person parameters range. With decreasing similarity, score transformation precision benefits more from good targeting. Difficulty shifts up to two logits somewhat increased the estimation bias but without affecting the transformation precision. The observed robustness against difficulty shifts supports the advantage of applying a true-score equating methods over identity equating, which was used as a naive baseline method for comparison. Finally, larger sample size did not improve the transformation precision in this study, longer scales improved only marginally the quality of the equating. The insights from the simulation study are used in a real-data example.

## Introduction

### Background

The process of equating aims to make scores issued by different multi-item scales comparable (Kolen & Brennan, 2014). Smith and Kramer (1992) divide the theoretical framework for test equating into two methodological approaches: observed-score and true-score equating.

Observed-score equating procedures apply a transformation to approximate the observed-score distributions from different scales. Typically, these procedures first use methods to reduce random variation in the raw scores through, for example, pre-smoothing, estimation of score probabilities, or item response theory (IRT) modeling (von Davier et al., 2013) and then equate the scores, for example, with linear, equipercentile (Skaggs & Lissitz, 1986), or kernel equating methods (Wiberg, 2016). Overviews of observed-score equating methodologies can be found in Peabody (2020) or Battauz (2017).

True-score equating also aligns scores from different scales. The so-called “true score” is established through a latent variable model (Kolen & Brennan, 2014). True-score equating uses one concurrent calibration of scales to estimate common item parameter ( δ ) within a single run and derive specific person parameter ( θ ) for each scale. When applicable, true-score equating has been found to be more accurate than observed-score equating (Chen et al., 2009; Hanson & Beguin, 2002; Peterson, 2007; Zhang et al., 2013). However, these findings are not consistent and depend on characteristics of the equating condition (Arai & Mayekawa, 2011; Lee & Ban, 2009; Tong & Kolen, 2005).

Several assessment designs are available to achieve score comparability through true-score equating. A concurrent calibration of scales can be applied in a common person design (horizontal design) or using a non-equivalent anchor test (NEAT) design. NEAT designs are used when test-takers have answered different scales with some common items, so-called anchor items. NEAT designs are considered more economical and practical than common person designs but also pose challenges, mainly in selecting the best anchor items (Diao & Keller, 2020; He & Cui, 2020; Suanthong et al., 2000). The concurrent calibration with a common person design is often found to be the most intuitive of the latent trait-based approaches with a robust linking approach that only requires all test-takers to answer the different scales (Dorans, 2007). The concurrent calibration analyses, two or more assessment scales simultaneously and ensures that the δ , that is, the item parameter, are put on the same measurement continuum. In the literature, 20 common dichotomously rated items with sample sizes of 100 showed the best true-score equating properties in Suanthong et al. (2000). K. F. Cook et al. (2007) recommend having sample sizes of at least 300 for equating multiple scales. A concurrent calibration is ideal when two scales are equated but becomes difficult and inefficient with more than two scales (Surla, 2020).

Equating with a common person design, also called form-to-form equating, is highly relevant in different fields of questionnaire-based measurement. In educational settings where test-takers can prepare for an examination with a “drill-and-practice” session before undergoing the actual exam, the different scales should be equivalent in terms of their difficulty (Almond et al., 2003). For health measurement, many different scales are available when deciding to measure a health or health-related aspect of interest (Ballert et al., 2019; Velstra et al., 2011). Equating and having a transformation equation enables harmonizing the information collected with different scales and having metrically sound scores for broader usage (Chan et al., 2015; Smith & Taylor, 2004). Equating also relates to item banking. An item bank consists of an item pool placed onto one underlying common metric. Different subsets of an item bank are expected to produce interchangeable metrics (Wolfe, 2000). Form-to-form equating is also relevant when creating a shorter version of an assessment tool. Equated test versions inform the elimination of items by preserving those which best transfer scores from the short to the original version (Ryan & Brockmann, 2010; Xiao et al., 2019).

The multi-item assessment context mimicked in this simulation study is motivated by health-related settings. Individual information about functioning, physical and mental health, as well as living conditions are getting more and more routinely assessed by health systems (ANQ, 2022; AROC, 2022; Canadian Institute for Health Information, 2022). Assessments in health settings are often of smaller scale and often within populations that need to present specific characteristics to be recruited, such as certain diagnoses or certain treatments. In health-related research, questionnaire-based assessments, particularly for studies, are voluntary. This typically leads to smaller sample sizes than in educational settings, for example. The multi-item scales applied in this assessment context are also often shorter and rarely exceed 20 items. These multi-item scales are generally responded to by means of Likert-type response options, indicating increasing endorsement of an item. In this context, equating of scales can provide a richer quantitative basis for empirical studies or facilitate the interchangeability of scores derived from different scales. Data collections can take place within one assessment session with an intention from the assessor to equate scales (Choi et al., 2014; Covic et al., 2012; Gatz et al., 2015; Maritz et al., 2022; Vos et al., 2019). Sometimes, data that are collected over time to monitor the course of a treatment or a rehabilitation intervention can be made available for scale equating (Edelen et al., 2021; Fischer et al., 2011; Jones et al., 2021; McDonough et al., 2020; Taylor & McPherson, 2007; ten Klooster et al., 2013; Velozo & Woodbury, 2011). In health research, equating studies using a common person design are often described by the terms “instrument crosswalk” or “data harmonization.”

In theory, equating is described as ideal when the requirements of equal construct, equal reliability, symmetry, equity, and population invariance are met (Peterson, 2007). By and large, two equated scales, for example, Scale X and Scale Y, should measure, at least to some extent, the same construct with the same reliability. The symmetry requirement expects that the transformation equation is bidirectional. Equity underlines that none of the scales should be more favorable to test-takers. Population invariance assumes that the equating function remains invariant to sample characteristics. Equating literature does not explicitly mention the quality of the scale targeting as a fundamental requirement for equating. However, the good targeting of a scale to the population is important and routinely tested and reported in psychometric studies. A well-targeted scale consists of items that match the ability continuum of the test-takers. Equating when one or all scales do not match a sample’s ability range is questionable and so worth examining. Targeting can be understood as an equity problem when scales are not assessing the same θ ranges. For simplicity, we will treat equity and targeting as two separate concepts, the first being interested in differences in the difficulty of scales and the second in the δ and θ parameter match. Targeting relates also to the notion of reliability, as the computation of the reliability in Rasch analyses is based on the dispersion of the θ estimates and their measurement error. This simulation, however, did not explicitly control for the reliability, so that the reliability will only be discussed as a corollary of the simulated targeting.

This study systematically observed the effects on score transformation precision when similarity of the constructs being assessed, their equity, and their targeting were varied. The requirement of equal constructs is central to the present study. In scale construction, the similarity of the latent trait assessed by scales using different items is rarely described as perfect. Disparate operationalizations of one latent construct can diminish the unidimensionality. For example, scales that examine algebra proficiency with *equation*-type and *story*-type exercises would typically lead to a multidimensional measure of algebra (Ackerman, 1994). Also, characteristics of the scales, such as the wording of the items (McCreary et al., 2013; Romine et al., 2018; Yamaguchi, 1997) or their response options (Bolt & Adams, 2017) can impact the dimensionality of a common metric. Regardless, if the researcher assumes unidimensionality, true-score equating will just treat the constructs as unidimensional. Statistically, the similarity of instruments’ content can be discussed in terms of the degree of multidimensionality and expressed, for example, by a factor correlation parameter when analyzed with a multidimensional approach. With higher correlation, two scales present more similar operationalizations of a latent trait (Reckase et al., 1988). Equating conditions are ideal with unidimensionality, with one unique latent construct being measured by the two scales. L. L. Cook and Eignor (1989) point out that statistical unidimensionality is not granted even when the same latent construct is assessed by both scales. Some studies indicate that equating with Rasch or IRT methods may still perform well despite departures from strict unidimensionality (Beguin & Bradley, 2001; Surla, 2020). Bolt (1999) found that with correlations between scales above 0.7, true-score equating performs better than observed-score approaches. For IRT-based equating, Choi et al. (2014) reported that for the equating of health outcome measures, inter-scale correlations of 0.75 to 0.8 might be an appropriate minimum when a common person design is used. Fischer et al. (2011) reported good scale score conversions using depression scales that had a correlation of 0.85.

In conjunction with the equal construct requirement, this simulation study also addressed the impact of departures from equity. Equity underlines that none of the scales should be more favorable to test-takers. It must be a matter of indifference whether test-takers assess scale X or scale Y (Lord, 1980), meaning that the scales should be equally difficult. If equity holds, scores for an individual should be identical regardless of the scale. When scales are not equally difficult and measure different ranges of the θ continuum, compliance to the equity requirement is not granted, and the precision of score transformations along the entire measurement continuum may be questioned.

This study also considered good targeting as an essential prerequisite when equating scales. Targeting is considered good when the δ from the scales to be equated match the θ distribution. It seems that simulation-based equating studies have not been very interested in observing effects of mismatch of the distribution of the θ relative to the δ distribution. If equating studies vary the δ or sometimes the θ distribution, then mostly to challenge the equity requirement, in the form of a shift of the mean, i.e., simulating change or growth, as in Kopp and Jones (2020), Han et al. (2012), He et al. (2013), or Waterbury and DeMars (2021). The skewness of the distribution of the θ parameter is also sometimes varied, but by preserving good targeting, that is, with overlapping dispersion of the δ and θ , as in Manna and Gu (2019) or Keller and Keller (2015). In any case, Suanthong et al. (2000) mention, citing L. L. Cook and Paterson (1987), that special care must be taken whenever the ability of groups assessed differ in level and dispersion.

This equating study uses concurrent calibration with the partial credit model (PCM), a Rasch-type model for polytomous response options (Masters, 1982, 1985). In the following, for simplicity, we will refer to both the Rasch model for dichotomous responses (Rasch, 1960) and extension of the Rasch model for polytomous responses (Masters, 1982) as “Rasch models.” Rasch models build on a probabilistic approach to measurement where the probability of a response to an item is formalized as a function of the item difficulty and the person ability parameters. Skaggs and Wolfe (2010) provide a comprehensive overview on the equating of scales with Rasch models and different equating designs. Smith and Kramer (1992) report that equating procedures are robust to violations of the assumptions of the Rasch model. In this study, the violations occurring were only those intentionally caused by the simulation.

Many true-score equating simulation studies focus on IRT, that is, two-parameter logistic (2PL) or higher-order models for dichotomous item ratings. Rasch-based simulation studies interested in equating are rarer. The effect of lack of similarity of scales in a common person design is very little investigated. This study aims to shed light on the accuracy of score transformations across scales equated through concurrent calibration with a Rasch model. Especially, we want to come up with some concrete insights about the effect of departures from scale similarity, equity, and targeting when equating with the PCM and using a common person design. Before diving into Rasch-based true-score equating, an Appendix is available as Online Supplemental Material for interested readers that describes the measurement assumptions of the PCM and the criteria applied in this study to determine whether the assumptions are met.

### True-Score Equating

The true-score equating approach in a common person design uses a process with distinct analysis steps to arrive at a score transformation rule that links the scores from the equated scales. The steps required to equate two scales, called Scale X and Scale Y, throughout the study are described in what follows and shown in Figure 3.

#### Separate PCMs

Preliminary to the equating of scales, it is essential to consider the degree to which items exhibit proper fit within the respective scales (Wolfe, 2000; Wright & Bell, 1984). An analysis with the PCM determines the extent to which data collected with the scales fulfill fundamental measurement assumptions (see Appendix A in the Supplemental Online Materials). However, separate calibrations of the two scales produce independent estimates of $\hat{\delta }$ that are linked to the measurement characteristics of the respective assessment scale, that is, ${\hat{\delta }}_{X}$ , ${\hat{\delta }}_{Y}$ . In this context, the total scores for Scale X, that is, $R_{X}$ , and for Scale Y, that is, $R_{Y}$ are understood to be equal to the respective expected raw score given the estimated level of ability of a person ( $\hat{\theta }$ ) and the estimated difficulty of the items $\hat{\delta }$ of Scales X or Y, that consist of a set of $I_{x}$ and $I_{y}$ items, respectively:

$$R_{X}=\Sigma_{i=1}^{I_{x}}E\left[X_{\mathrm{ni}}\right]=\Sigma_{i=1}^{I_{x}}\Sigma_{x_{i}=0}^{m_{i}}\mathrm{Pr}\left(X_{n_{i}};{\hat{\theta }}_{X},{\hat{\delta }}_{X}\right),$$

for form X, and

$$R_{Y}=\Sigma_{i=1}^{I_{y}}E\left[X_{\mathrm{ni}}\right]=\Sigma_{i=1}^{I_{y}}\Sigma_{x_{i}=0}^{m_{i}}\mathrm{Pr}\left(X_{n_{i}};{\hat{\theta }}_{Y},{\hat{\delta }}_{Y}\right),$$

for form Y. The $X_{\mathrm{ni}}$ represents the observed response of nth person on the ith item that has 1 to $m_{i}$ response options.

The ${\hat{\theta }}_{X}$ and ${\hat{\theta }}_{Y}$ values are not exchangeable across the scales as they are issued by separate analyses and linked to the difficulties, ${\hat{\delta }}_{X}$ and ${\hat{\delta }}_{Y}$ specific to each scale. An equating model is necessary to establish the metric equivalence of the scales (L. L. Cook & Eignor, 1989).

#### Concurrent PCM

In this study design, where all test-takers have filled all different scales to be equated, the equating strategy can consist of a joint calibration of all the $I_{\mathrm{xy}}=I_{x}+I_{y}$ items, with one measurement model. The so-called concurrent calibration results in a common measurement continuum by conditioning the estimation of the δ and consequently θ parameters on the total scores of the two scales. The combined total score, $R_{\mathrm{XY}}$ , with $R_{\mathrm{XY}}=R_{X}+R_{Y}$ , is a sufficient statistic to estimate equated ${\hat{\delta }}_{\mathrm{XY}}$ for the two scales. The total scores can be formalized here also as the product of the item response probabilities given a common ${\hat{\theta }}_{\mathrm{XY}}$ across items with difficulty ${\hat{\delta }}_{\mathrm{XY}}$ , that is,

$$R_{\mathrm{XY}}=\Sigma_{i=1}^{I_{\mathrm{xy}}}E\left[X_{\mathrm{ni}}\right]=\Sigma_{i=1}^{I_{\mathrm{xy}}}\Sigma_{x_{i}=0}^{m_{i}}\mathrm{Pr}\left(X_{\mathrm{ni}};{\hat{\theta }}_{\mathrm{XY}},{\hat{\delta }}_{\mathrm{XY}}\right).$$

The ${\hat{\delta }}_{\mathrm{XY}}$ parameter from the concurrent calibration is not identical to the ${\hat{\delta }}_{X}$ and ${\hat{\delta }}_{Y}$ of the separate calibration in Step 1. In this study, the concurrent calibration included all the data from Scale X and Scale Y, which were analyzed with a unidimensional PCM, independently of the degree of association between the scales.

#### Anchored PCMs

The concurrent calibration in Step B equates Scale X and Scale Y and puts the two metrics on a common measurement continuum. ${\hat{\theta }}_{\mathrm{XY}}$ is an estimate of θ based on the common calibration of both scales. As for the separate calibration, we now use ${\hat{\theta }}_{X}$ to denote a θ estimate based on the responses given to Scale X, and correspondingly ${\hat{\theta }}_{Y}$ to denote a θ estimate based on the responses given to Scale Y. However, in the anchored analysis, the item parameter of each scale is fixed to the item parameter values ( ${\hat{\delta }}_{\mathrm{XY}}$ ) obtained through concurrent calibration. This leads to the equation:

$$R_{X}=\Sigma_{i=1}^{I_{x}}E\left[X_{\mathrm{ni}}\right]=\Sigma_{i=1}^{I_{x}}\Sigma_{x_{i}=0}^{m_{i}}\mathrm{Pr}\left(X_{n_{i}};{\hat{\theta }}_{X},{\hat{\delta }}_{\mathrm{XY}}\right).$$

for the anchored form X, and

$$R_{Y}=\Sigma_{i=1}^{I_{y}}E\left[X_{\mathrm{ni}}\right]=\Sigma_{i=1}^{I_{y}}\Sigma_{x_{i}=0}^{m_{i}}\mathrm{Pr}\left(X_{n_{i}};{\hat{\theta }}_{Y},{\hat{\delta }}_{\mathrm{XY}}\right).$$

for the anchored form Y.

The process of preliminary fixing the δ parameters of the scales is called anchoring or also constraining. The separate calibrations using the anchored item estimates ( $\delta_{\mathrm{XY}}$ ) adopted from the common calibration will ensure that the derived $\hat{\theta }$ are on the common metric, that is, ${\hat{\theta }}_{X}\equiv {\hat{\theta }}_{\mathrm{XY}}\equiv {\hat{\theta }}_{Y}$ . Equal values of ${\hat{\theta }}_{X}$ and ${\hat{\theta }}_{Y}$ then indicate an exact same level of ability because they stem from the same measurement continuum (Paek & Cole, 2019).

#### Concordance Table

When considering a latent trait being measured, each test-taker is expected to have only one ability level. Still, their test scores can vary because of the characteristics of the test versions used for the assessment. The concurrent calibration and analysis with anchored item estimates puts different scales on a common metric. A three-step score transformation is necessary to find the score on Scale Y that is the most equivalent to a score on Scale X. First, for a person n given raw score $R_{X_{n}}$ on Scale X, the corresponding anchored person parameter, ${\hat{\theta }}_{X_{n}}$ is determined. Second, the ${\hat{\theta }}_{Y}$ that corresponds closest to that ${\hat{\theta }}_{X_{n}}$ is found. Finally, the raw score on Scale Y, $R_{Y}$ , associated with the closest ${\hat{\theta }}_{Y}$ is looked up. Consequently, the closest common person parameter estimate, ${\hat{\theta }}_{X}\equiv {\hat{\theta }}_{Y}$ , indicate most similar levels of ability and allow to link the most equivalent raw scores, $R_{X}$ and $R_{Y}$ , from the two scales. An approximation is often used to find the most equivalent ${\hat{\theta }}_{X}$ and ${\hat{\theta }}_{Y}$ estimates across equated scales and requires searching for $\mathrm{argmi}n_{x}f\left(x\right):=\{x\in {\hat{\theta }}_{X_{n}}:f\left(x\right)=\left|{\hat{\theta }}_{X_{n}}-{\hat{\theta }}_{Y}\right|\}$ . In practice, the functional relationship between two scale scores via the $\hat{\theta }$ estimates is often summarized by a so-called concordance table that directly links the equated raw test scores. A concordance table has a user-friendly format that easily links a score observed on Scale X to the equated score on Scale Y (ten Klooster et al., 2013). Table 1 gives one example of how a concordance table can be set up. Sometimes, the logit-scaled estimates of the $\hat{\theta }$ and their measurement error are also shown. Sometimes the logit-scaled estimates are rescaled to represent an accessible reference metric (e.g., a 0–100 scale) instead of the raw score ranges. For the example in Table 1, the raw scores and their transformed scores were obtained with Scale X and Scale Y having a small factor correlation of $\rho_{\mathrm{sim}}=0.25$ but good targeting and no difficulty shift. As Scale X and Scale Y have the same score range from 0 to 20, the first column contains the values of the observed score for both scales, and the remaining two columns the respective transformed scores. For a transformation of a Score X toward the expected score on Y, the score of X has to be found in the first column, and the transformed score looked up under the expected scores on Y. For example, if the score on Scale X is 12, the expected score on Scale Y would be a 10, but for a score of 12 on Y the expected score on Scale X would be a 15.

**Table 1.** Example of a Concordance Table.

Raw	Equated	Equated
Scores	Scores	Scores
X or Y^a^	X	Y
0	0	0
1	2	1
2	3	1
3	4	2
4	5	3
5	7	4
6	8	5
7	9	5
8	10	6
9	11	7
10	12	8
11	14	9
12	15	10
13	16	11
14	16	11
15	17	12
16	18	13
17	19	15
18	19	16
19	20	17
20	20	19

a Here with two instruments having the same score range from 0 to 20.

After equating, the concordance table directly links observed scores in one scale to the expected score on an equated scale. Applied psychometric equating studies using the Rasch model routinely report the respective fit of the scales to be equated to the Rasch model, often also the fit of the equated test versions to the Rasch model, and typically provide a user-friendly score concordance table that should facilitate score transformations in the applied work field (Chen et al., 2009; Doğanay Erdoğan et al., 2017; Lambert et al., 2015; Latimer et al., 2012).

### Aims of the Study

This simulation study hopes to contribute to the methodological literature in the field of true-score equating with the Rasch model and to provide some insights into the impact of characteristics of the scales on score transformations. The general objective of the study is to use simulated data to investigate the precision of score transformations based on Rasch-based equating in a common person design when varying the degree of similarity, the degree of equity, and the targeting of scales.

Concordance tables allow converting individual raw scores from one scale to the other. Data from a common person design, where all test-takers answered all equated scales, permit checking the transformation quality directly. For example, it is possible to directly determine for each simulated test-taker whether a transformation of a raw score on Scale X ( $R_{X}$ ) to its expected score on Scale Y ( ${\hat{R}}_{Y}$ ) comes close to what can be observed in the data, that is, the true raw score found in Scale Y ( $R_{Y}$ ), and quantify the discrepancy. One possible statistic for describing the degree of discrepancy between the observed and the expected score is the mean squared error (MSE), in other words, the mean of the squared differences between the transformed raw score and the observed raw score, $\frac{1}{N}\sum_{n=1}^{N}{\left(\underset{n}{\hat{R}}-R_{n}\right)}^{2}$ . The root of the MSE (RMSE) is a more commonly reported as metric for the precision that is formalized as:

$$\mathrm{RMSE}=\sqrt{\frac{1}{N}\sum_{n=1}^{N}{\left({\hat{R}}_{n}-R_{n}\right)}^{2}}.$$

However, the possible values of the RMSE depend on the assessment range, for this reason the normalized RMSE (NRMSE) is reported, that normalizes the RMSE by the score range of the scales:

$$\mathrm{NRMSE}=\frac{\mathrm{RMSE}}{R_{\max}-R_{\min}}.$$

The bigger the NRMSE, the higher the discrepancy between the score obtained through true-score equating ( $\hat{R}$ ) and the true raw score ( R ) of a scale, and the lower the transformation precision. The NRMSE is often interpreted as a percentage, here of the overall scale range (Otto, 2019). In this simulation study, the transformation precision is reported bidirectionally, from Scale X to Scale Y and reversely from Scale Y to Scale X; because in some simulation settings, only Scale Y has a higher dispersion of the θ parameters.

In addition to the NRMSE, which is composed of bias and variance together, the percent bias is also reported separately. The percent bias ( %Bias ) indicates the proportion of the transformation precision that is not explained by the variance of the scores. The MSE can be decomposed into the variance of the score and a bias, that is, $\mathrm{MSE}=\mathrm{Va}r_{\hat{R}}+{\mathrm{Bias}}_{\hat{R}}^{2}$ . The bias of the transformed score is formalized as $\mathrm{Bia}s_{\hat{R}}=\frac{\Sigma_{n=1}^{N}\left(R-\hat{R}\right)}{N}$ , that is, the mean difference between the observed and the transformed scores. The size of the bias is affected by the scale length or score range. The percent bias is then formalized as $\%\mathrm{Bias}=\frac{\Sigma_{n=1}^{N}\left(R-\hat{R}\right)}{\Sigma_{n=1}^{N}R}*100$ . The percent bias can indicate positive or negative bias. Forero and Maydeu-Olivares (2009) recommend treating absolute percent bias values below 10% as negligible, values of 10% to 20% as substantial, and >20% as unacceptable.

Furthermore, the improvement in the quality of the score transformation using the equated model is investigated by comparing it to an identity equating. Identity equating reflects an ideal situation where no equating would be necessary to compare the scores of two tests. Identity equating is based on the strong assumption that a score on a Scale X is equivalent to the same score value on a Scale Y. Identity equating was added to this analysis as a baseline for comparison and to highlight the advantage that Rasch-based true-score equating brings.

The findings of the simulation study are illustrated by means of an empirical example using real data from the World Health Organization’s and World Bank’s Model Disability Survey (MDS) in Afghanistan in 2019 (Sabariego et al., 2022; Shinwar et al., 2020). In this example, the psychometric characteristics of two set of items are related to the closest simulation setting to discuss the observed and expected transformation precision after equating.

## Simulation Study

### Data Generating Process

This simulation study was carried out with R 4.0.3 (R Core Team, 2020). The data were simulated with the function *simdata()* from R package *mirt* for the simulation of uni- and multidimensional data for IRT models (Chalmers, 2012).

**Figure 1.**
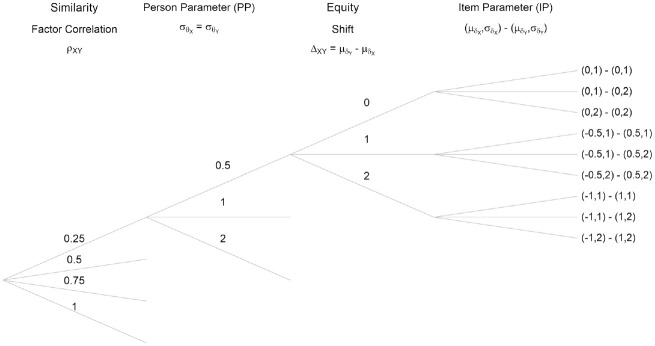
Configuration of the Simulation Parameters, Identically Repeating for All Started Branches, and Repeated for Sample Sizes 500 and 1,000, for 10 and 20 Item Scales.

Figure 1 summarizes the combinations of the different simulation settings, specifically the parameters that were varied in this study. Only the upper part of the figure is explicitly laid out, but the simulation scheme repeats identically for each started branch. These simulation settings were repeated for sample sizes N=500 and N=1000 and scale lengths of I=10 and I=20 polytomous items with three response options. This results in a total of 4×3×3×3×2×2=432 different simulation settings, with four degrees of correlation ( $\rho_{\mathrm{sim}}$ ), three θ dispersions ( $\sigma_{\theta }$ ), three item parameter shifts ( $\Delta_{\mathrm{XY}}$ ), three δ -dispersions ( $\sigma_{\delta }$ ), two sample sizes (N), and two scale lengths (I). These 432 different settings were repeated 500 times each to control for the random variation in the data generation. A polytomous instead of a dichotomous response type was chosen, as scales used in health and health-related research more typically present Likert-type response options that assess the degree of endorsement to an item. Dichotomous ratings of type of yes/no, true/false, or correct/incorrect are more typical for educational assessments. Scales we had in mind when designing the study were for example mental health scales such as the Psychological Health Questionnaire with 9 items rated by means of 4 response options, the Mental Health Index with 5 items and 6 response options, or the Hospital Anxiety and Depression Scale with 14 items and 4 response options. A posteriori rescoring of items to reduce the number of options, so-called collapsing of response options, is rather common also for well-established scales (C. J. Gibbons et al., 2011; Lin et al., 2017; Vilca et al., 2022; Zhong et al., 2014).

When simulating data with *simdata()* it is possible to control for characteristics of the model, including item discrimination (via argument *a*), item difficulty thresholds (via argument *d*) as well as their mean difficulty (via argument *mu*), the sample size (via argument *N*), and the type of items simulated, e.g., dichotomous or polytomous (via argument *itemtype*). This study used the *itemtype = “gpcm,”* that is, a generalized PCM parameterization, but by constraining slope parameters to equal 1, as the generating of multidimensional data is not directly available for Rasch models in *mirt*.

The different degrees of similarity between scales were operationalized by varying the degree of multidimensionality (more pronounced multidimensionality going along lower degrees of similarity). Unidimensionality across scales indicates perfect similarity. Simulation of the similarity of the scales is closely related to the simulation of the person parameter. The person parameters ( θ ) distribution is controlled by the *simdata()* arguments *theta* or *sigma*. The argument *theta* permits the direct input of a vector of logit-scaled θ values of the desired length ( N ) and distribution. The argument *sigma* also permits simulating θ , but by entering a covariance matrix that controls for the underlying θ -distributions and the similarity of the θ distributions across the two dimensions. The diagonal of the covariance matrix entered in *simdata* option *sigma* represents the dispersion or variance of the θ parameters in each dimension. The off-diagonal elements of the matrix are the covariances. The size of the covariance relative to the variance determines the correlation between the two dimensions, or scales, as these two statistics are directly related:

$$\rho_{\theta_{X}\theta_{Y}}=\frac{\sigma_{\theta_{X}\theta_{Y}}}{\sigma_{\theta_{X}}\sigma_{\theta_{Y}}}.$$

The correlation, $\rho_{\theta_{X}\theta_{Y}}$ , of two ability vectors $\theta_{X}$ and $\theta_{Y}$ , for Scale X and Y , respectively, is a function of the covariance between $\theta_{X}$ and $\theta_{Y}$ ( $\sigma_{\theta_{X}\theta_{Y}}$ ) and the product of their standard deviations $\sigma_{\theta_{X}}\sigma_{\theta_{Y}}$ .

In what follows, the notation $\rho_{\mathrm{sim}}$ instead of $\rho_{\theta_{X}\theta_{Y}}$ is used when referring to the input for the simulation. The similarity of the scales was simulated as perfect ( $\rho_{\mathrm{sim}}=1$ ), large ( $\rho_{\mathrm{sim}}=0.75$ ), moderate ( $\rho_{\mathrm{sim}}=0.5$ ), and small ( $\rho_{\mathrm{sim}}=0.25$ ). For the multidimensional data generation, with correlations $\rho_{\mathrm{sim}}<1$ , the covariance values for the Scales X and Y were entered as:

$$\sigma_{\theta_{X}\theta_{Y}}=\rho_{\mathrm{sim}}\sigma_{\theta_{X}}\sigma_{\theta_{Y}}.$$

The θ dispersions for Scale X and Scale Y were equal within each setting and were set to $\sigma_{\theta }=0.5$ , $\sigma_{\theta }=1$ , and $\sigma_{\theta }=2$ , respectively.

The simulation study also challenged the equity assumption and investigated the impact of difficulty differences on the equating of scales in a standardized setting. To do so, item parameters ( δ ) were sampled by controlling first for the mean difficulty of the scale ( $\mu_{\delta }$ ) and the general dispersion ( $\sigma_{\delta }$ ) of the item parameter ( δ ). The mean difficulty, $\mu_{\delta }$ , of a scale refers to the mean difficulty across the I items of a test and can be formalized as

$$\mu_{\delta }=\frac{1}{\mathrm{Ik}}\Sigma_{i=1}^{I}\Sigma_{h=1}^{k}\delta_{\mathrm{ik}},$$

the dispersion refers to the standard deviation of the item difficulties:

$$\sigma_{\delta }=\sqrt{\frac{1}{I-1}\Sigma_{i=1}^{I}{\left(\delta_{i}-\mu_{\delta }\right)}^{2}}.$$

The dispersion of the difficulties of the items around the mean was set either to $\sigma_{\delta }=1$ or $\sigma_{\delta }=2$ for both scales or to $\sigma_{\delta_{X}}=1$ and $\sigma_{\delta_{Y}}=2$ when the measurement range differed across scales. For the simulation in a unidimensional setting, the item locations, that is, the average item difficulties, were drawn randomly from a normal distribution for both scales, respectively, with the setting specific mean and dispersion. As items were simulated to represent a polytomous scale with three response options, the two thresholds of each item ( $\delta_{\mathrm{ik}}$ ) were positioned at equidistance from the item location, at a distance selected randomly from a normal distribution with mean=0.7 logits and SD=0.15 . The distribution of the difficulty thresholds generated through this parameterization had good face validity and approximated the expected differences in the difficulty levels of the simulated scales well. The simulation presented 3 degrees of mean item difficulty shifts between Scale X and Scale Y ( $\Delta_{\mathrm{XY}}=\mu_{\delta_{X}}-\mu_{\delta_{Y}}$ ). The mean item difficulties were shifted symmetrically around zero with $\Delta_{\mathrm{XY}}=0$ when $\mu_{\delta_{X}}=0$ and $\mu_{\delta_{Y}}=0$ , $\Delta_{\mathrm{XY}}=1$ logit when $\mu_{\delta_{X}}=-0.5$ and $\mu_{\delta_{Y}}=0.5$ , and $\Delta_{\mathrm{XY}}=2$ logits when $\mu_{\delta_{X}}=-1$ and $\mu_{\delta_{Y}}=1$ . For data generation following the multidimensional model, the mean difficulty of the scale can be specified with the argument *mu* of the *simdata()* function.

**Figure 2.**
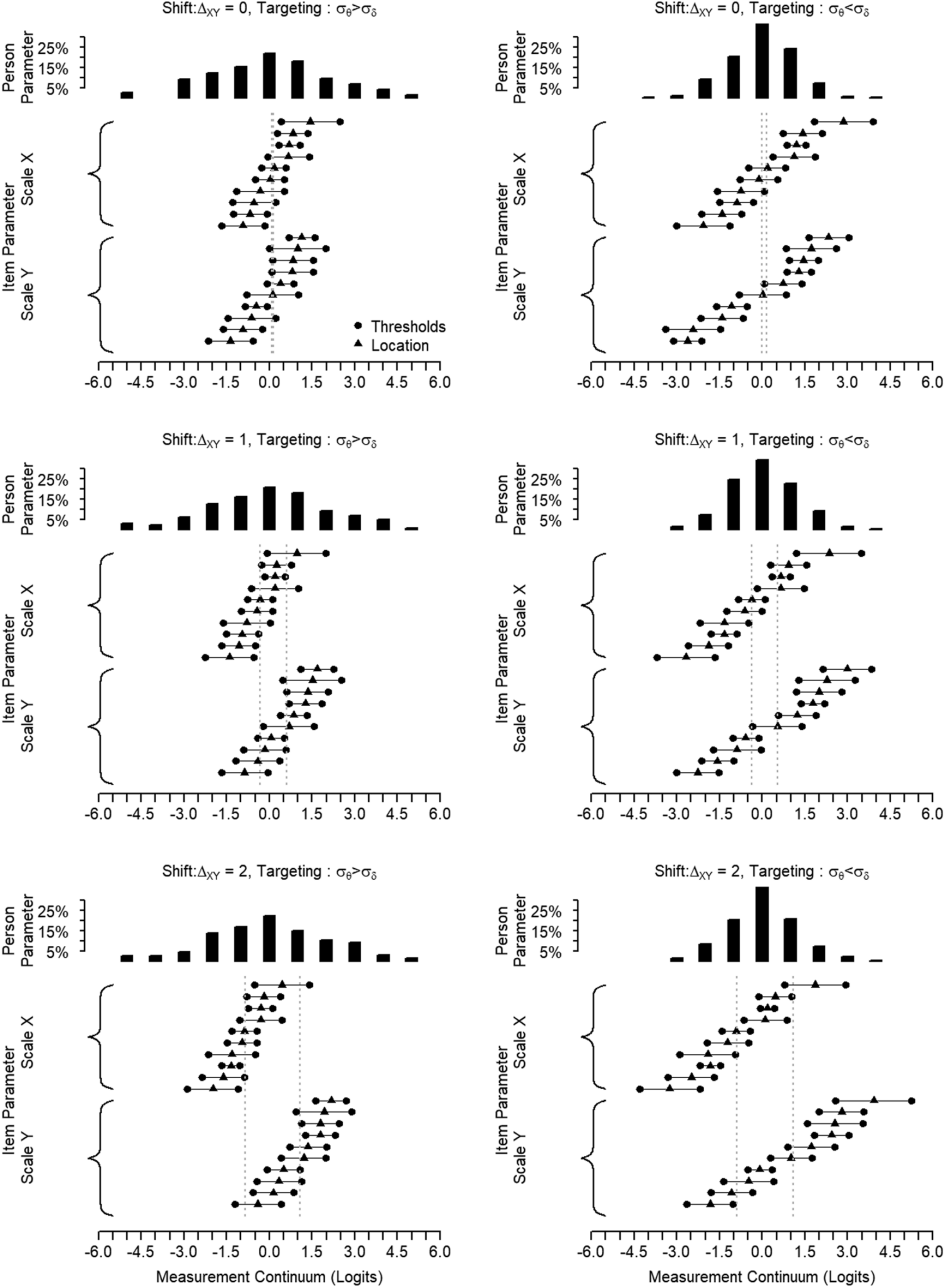
Example to Illustrate Targeting and Shift.

Figure 2 provides a visualization to understand better how the varying interplay of equity and targeting alters the match between δ and θ parameters from the concurrent calibration of the two scales. This is not shown for all settings, but when the dispersion of the θ (i.e., $\sigma_{\theta }$ ) is larger ( > ) or smaller ( < ) than the dispersion of the δ (i.e., $\sigma_{\delta }$ ), with increasing difficulty shift( $\Delta_{\mathrm{XY}}=0$ , $\Delta_{\mathrm{XY}}=1$ , $\Delta_{\mathrm{XY}}=2$ ) and for sample size *N* = 500 and scales with 10 items each. For each separate figure on the panel, the upper part shows the distribution of the θ and the lower part the δ distribution for Scale X and Scale Y, respectively. The gray dotted vertical lines are positioned at the mean of the δ parameter for each respective scale. They overlap for settings with no simulated difficulty shift and increase in distance for settings with increasing scale difficulty shift.

**Figure 3.**
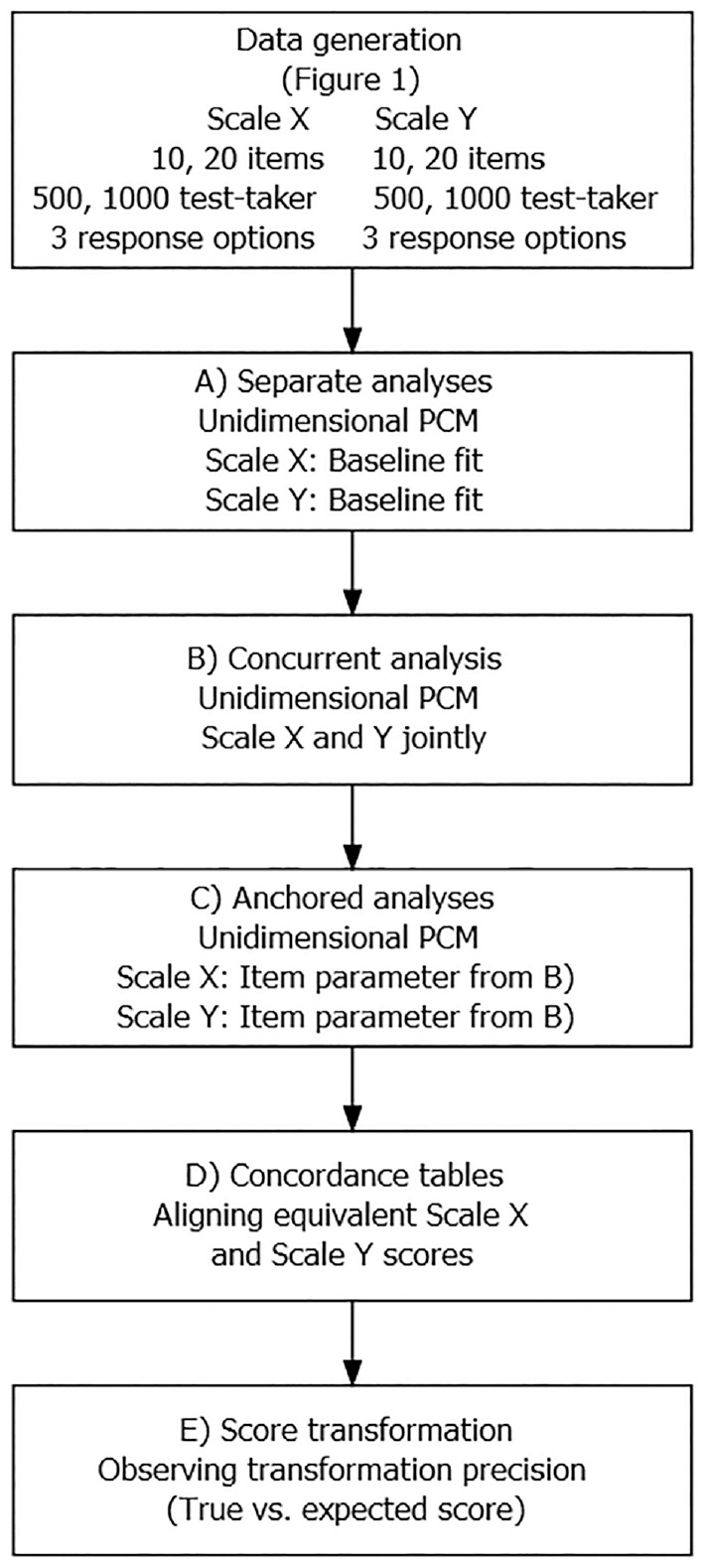
Flowchart for the Analysis Steps in One Simulation Setting. *Note.* PCM = partial credit model.

The different steps of the study, from the data-generating process to the final score transformation analysis, are schematically depicted in Figure 3. The most relevant lines of the R-code for the data generation and the true-score equating are provided in Appendix B in the Supplemental Online Materials.

## Results

**Figure 4.**
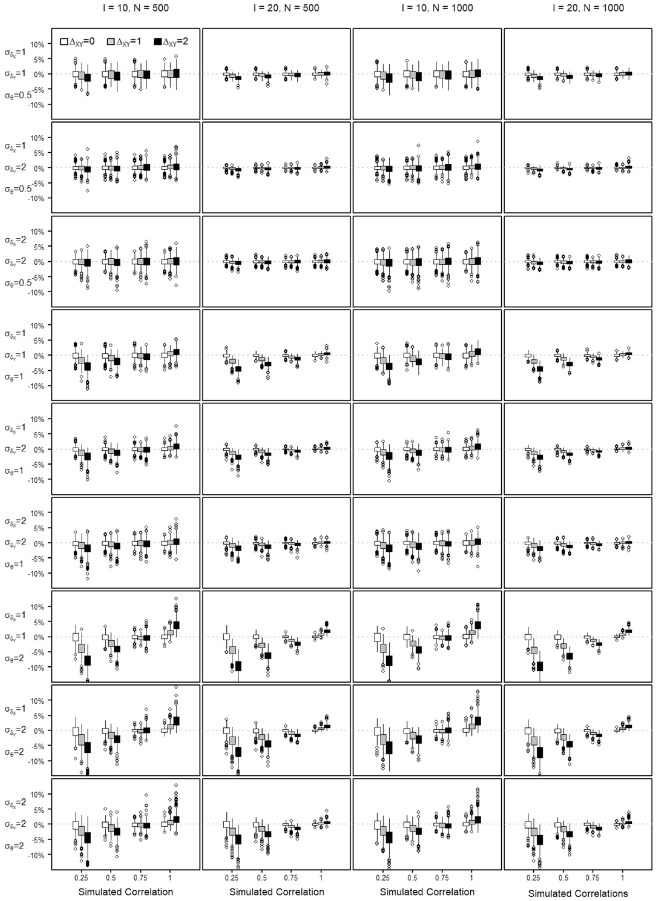
Percent Bias in Estimating the Score Transformation Precision From Scale X to Scale Y Across All Simulated Settings.

**Figure 5.**
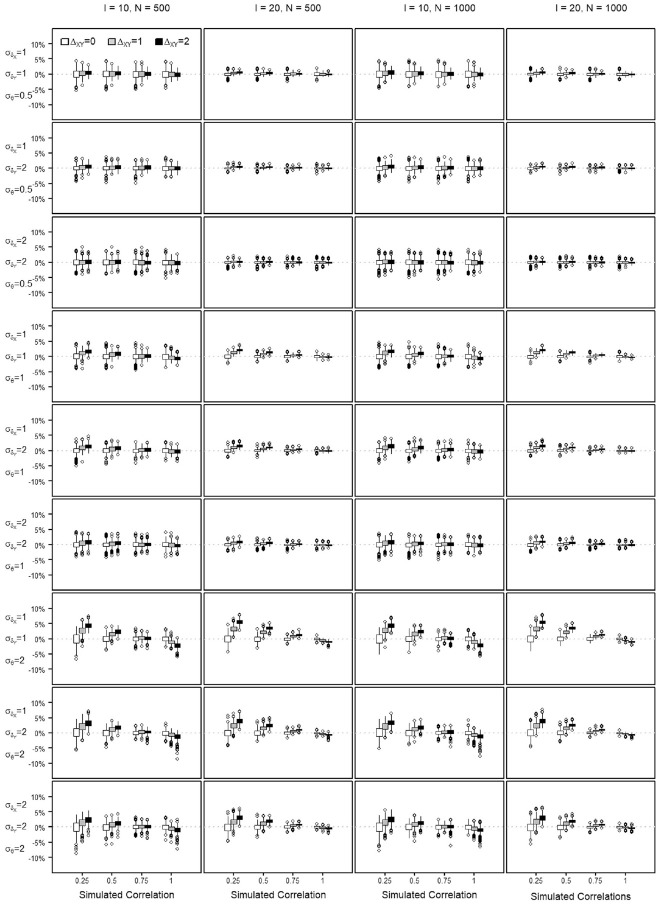
Percent Bias in Estimating the Score Transformation Precision From Scale Y to Scale X Across All Simulated Settings.

**Figure 6.**
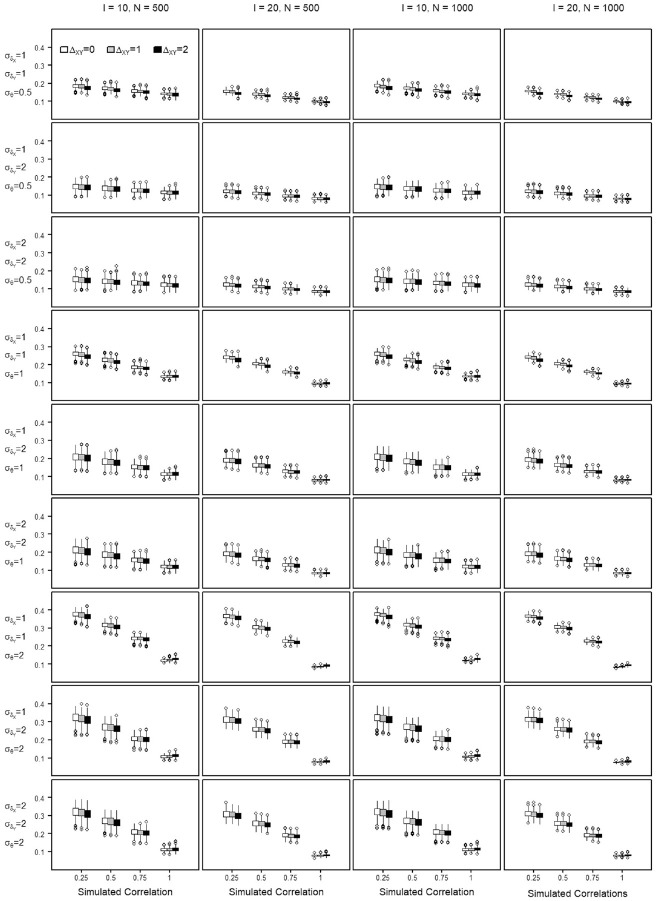
Normative Root Mean Square Error (NRMSE) in Estimating the Score Transformation From Scale X to Scale Y Across All Simulated Settings.

**Figure 7.**
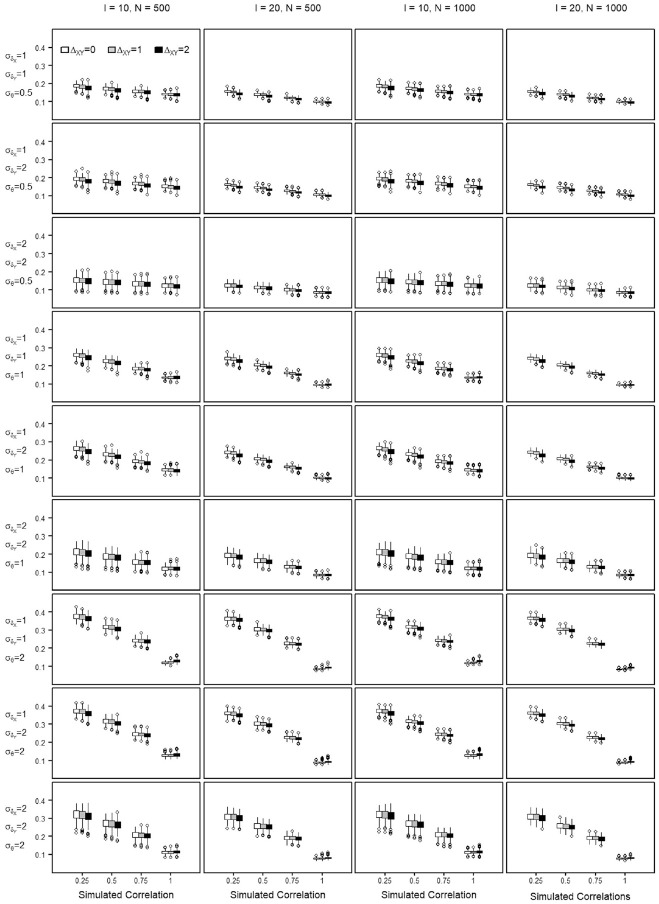
Normative Root Mean Square Error (NRMSE) in Estimating the Score Transformation From Scale Y to Scale X Across All Simulated Settings.

The percent bias shows the percentage of transformation precision that is not attributable to the variance of the score. The NRMSE is used to evaluate the precision of a score transformation between scales and quantifies the discrepancy between the equated score value and the true score of a scale. The distribution of the percent bias and the NRMSE values obtained across the repetitions of each simulation setting are summarized by means of boxplots in Figures 4 to 7, when transforming from Scale X to Y and reversely. Each Figure summarizes the findings regarding the percent bias and the NRMSE by means of 9×4 panels, where the 4 panel columns are for the sample and scale size combinations, and the 9 rows of panels for the $\sigma_{\theta }$ , $\sigma_{\delta_{x}}$ , and $\sigma_{\delta_{Y}}$ combinations. Within each panel, boxplots for an increasing degree of shift are shown next to each other, 4 times, for each degree of simulated similarity. The values of the 25% and 75% quantiles for the percent bias and the NRMSE are presented in Appendix C in the Supplemental Online Materials per sample size and scale length setting in Table C.4 to Table C.7 and Table C.8 to Table C.11 respectively.

All quantiles for observed percent bias were below 10%, as seen in Figures 4 and 5. The figures show that the estimation bias is larger with less similar scales, especially when the dispersion of the δ parameters is not encompassing the dispersion of the θ parameters, that is, smaller correlation $\rho_{\mathrm{sim}}$ and $\sigma_{\delta }<\sigma_{\theta }$ . When $\sigma_{\delta }<\sigma_{\theta }$ , the size of the scale also affects the estimation bias, with more bias in the shorter, that is, I=10 , scales. The difficulty shift affected the estimation negatively, with increased negative (X to Y) or positive (Y to X) bias when equating scales with a bigger difference in the general difficulty.

In what follows, the equating precision when transforming from Scale X to Scale Y is written as $\mathrm{NRMS}E_{X\to Y}$ , and as $\mathrm{NRMS}E_{Y\to X}$ when transforming from Scale Y to X. The sample sizes and scale lengths did barely affect the score transformation precision, it is only marginally better with scale lengths of *I* = 20 (see Appendix C in the Supplemental Online Materials, Table C.8 to Table C.11 for the quantile values of the NRMSE). Across all settings, a higher similarity of the scales goes along with lower NRMSE, that is, higher equating precision. The precision improves almost linearly with increasing similarity (Figures 6 and 7), but the magnitude of the improvement varies for the different settings. With scales of perfect similarity, the effects of poor targeting and departures from equity are smaller.

In Figure 6, at $\rho_{\mathrm{sim}}=1$ , shown by the rightmost boxplots in all panels, all NRMSE values lie between 0.1 and 0.16 for the 10-item scales, and 0.07 and 0.11 for the 20-item scales. In other words, with perfect similarity of two scales, the average departure represents 10% to 16% of the score range of the 10-item scales, that is, an average departure of 2 to 3.2 points from the true score. For the 20-item scales, the average departure represents 7% to 11% of the score range, that is, 2.8 to 4.4 points. With the smallest similarity, that is $\rho_{\mathrm{sim}}=0.25$ , the loss in precision is most dramatic when the θ parameter dispersion was largest, that is, $\sigma_{\theta }=2$ . Independently of the scale length, with $\sigma_{\theta }=2$ and $\rho_{\mathrm{sim}}=0.25$ , most NRMSE values lie within 0.29 and 0.39, meaning an average departure of 5.8 to 7.8 points for 10-item scales and 11.6 to 15.6 points for the 20-item scales.

**Figure 8.**
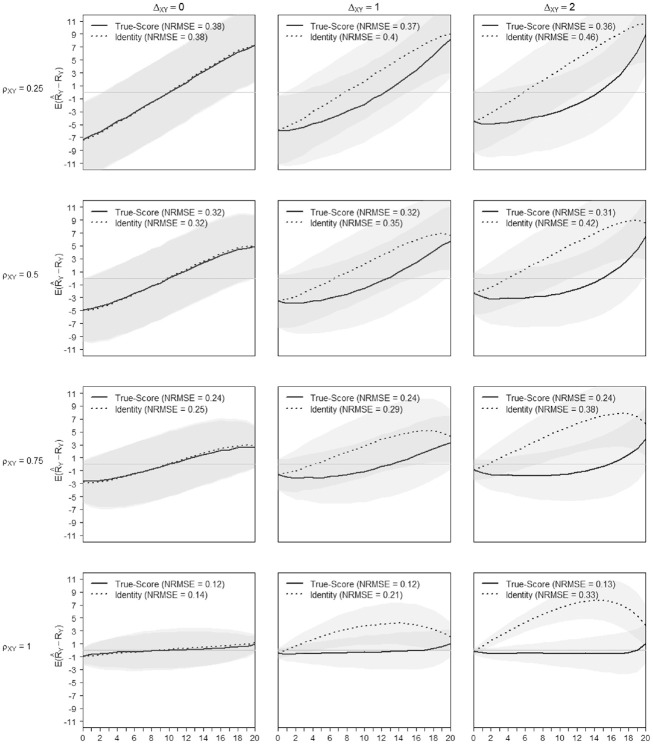
True-Score Versus Identity Equating of Scale X to Scale Y When $\sigma_{\delta_{X}}$ = $\sigma_{\delta_{Y}}$ = 1 and $\sigma_{\theta }$ = 2. *Note.* NRMSE = normative root mean square error.

**Figure 9.**
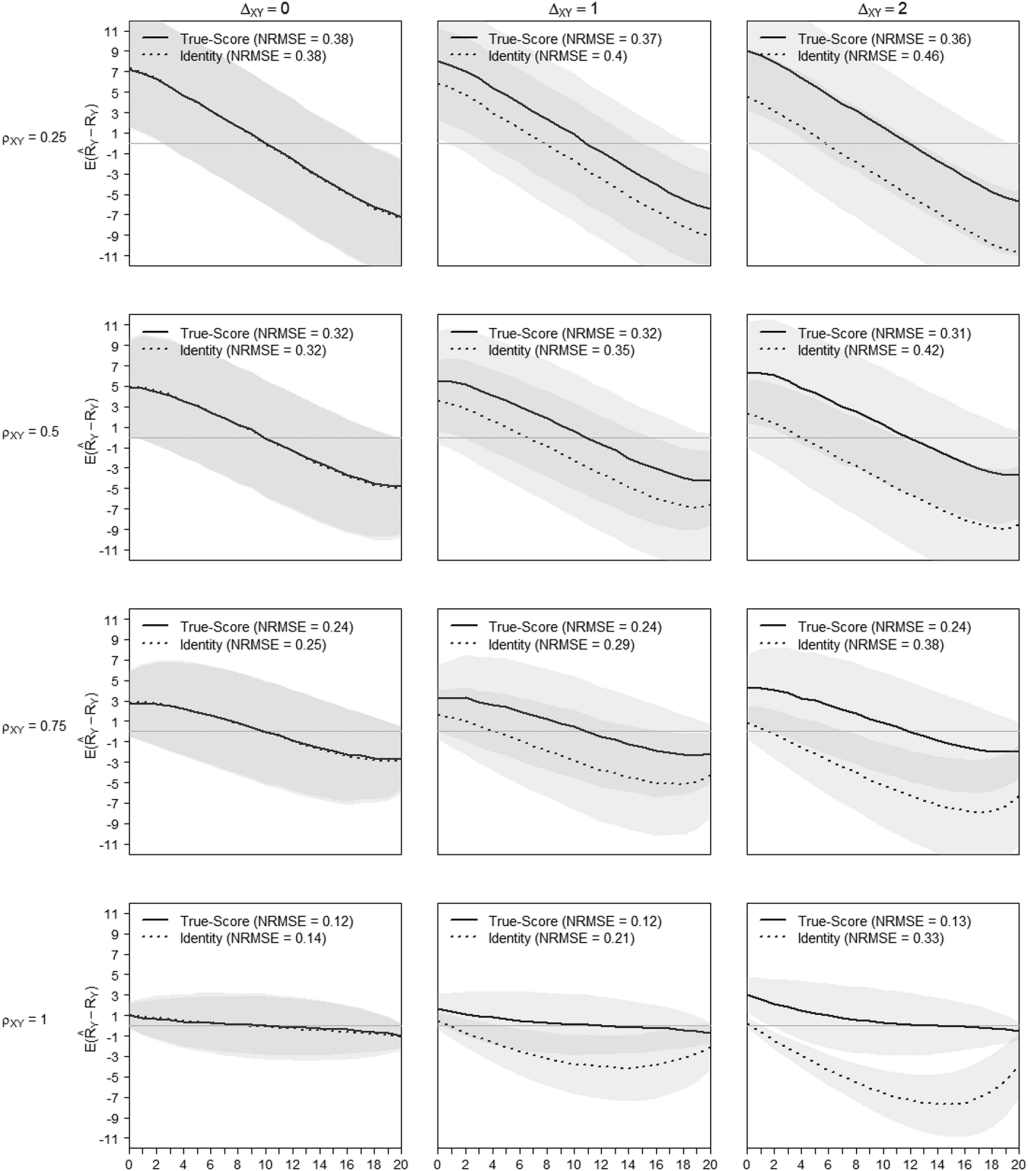
True-Score Versus Identity Equating of Scale Y to Scale X When $\sigma_{\delta_{X}}$ = $\sigma_{\delta_{Y}}$ = 1 and $\sigma_{\theta }$ = 2. *Note.* NRMSE = normative root mean square error.

Of course talking about the average departure in the score transformation precision is a rough simplification. It is well known that the precision is not uniformly distributed across the score range of a scale but increases toward the extremes (as will be illustrated in Figures 8 and 9 in this regard).

The positive effect of the high construct similarity on the precision of the score transformation is somewhat damped when transforming from settings with poor item-to-person targeting, where the δ parameters are not encompassing the θ space. With decreasing similarity of the equated scales, the negative effect of departures from good targeting increases steeply. With good targeting, the negative effect of decreasing construct similarity is less salient. This simulation supports a moderating effect with improved transformation precision across the similarity continuum when the δ distribution matches or spreads wider than the θ continuum ( $\sigma_{\delta }\geq \sigma_{\theta }$ —the first six rows of plots in Figures 6 and 7). In that sense, equating is more successful when similar scales can comprehensively capture the ability or proficiency spectrum of a population. The smaller the correlation, the more equating precision is gained with better targeting. When the measurement scope cannot cover the θ continuum ( $\sigma_{\delta }<\sigma_{\theta }$ ) (Row 7 plots in Figures 6 and 7), the precision of the transformation is lowest with NRMSE values close to 0.4, that is, average score departures close to 40% of the scale range. Higher equating precision is also systematically observed when transforming from a scale with smaller δ parameter dispersion ( $\sigma_{\delta_{X}}=1$ ) toward a larger one ( $\sigma_{\delta_{Y}}=2$ ), for example, with factor correlation $\rho_{\mathrm{sim}}=0.25$ and $\sigma_{\theta }=0.5$ , the 25% and 75% quantiles of the $\mathrm{NRMS}E_{X\to Y}$ are Q1 = 0.13 and Q3 = 0.16 and $\mathrm{NRMS}E_{Y\to X}$ are Q1 = 0.19 and Q3 = 0.2 for a test length of *I* = 10. For a test length of *I* = 20, the transformation improves, the 25% and 75% quantiles of the $\mathrm{NRMS}E_{X\to Y}$ being Q1 = 0.11 and Q3 = 0.13 and $\mathrm{NRMS}E_{Y\to X}$ are Q1 = 0.16 and Q3 = 0.17. Transforming from smaller to larger δ dispersion is always better, as illustrated by the change in position of the boxplots in panel rows 2, 5, and 8 when compared across Figures 6 and 7.

The NRMSE values are generally indicating better transformation precision with ability dispersions $\sigma_{\theta }=0.5$ than with $\sigma_{\theta }=1$ or $\sigma_{\theta }=2$ . Although the transformation appears more accurate with smaller θ -dispersion within the difficulty range of the scales, it needs first to be mentioned that the person separation reliability of these models was not acceptable. The person separation is smaller when the θ values are in a narrow range of the measurement continuum and cannot discriminate well among ability levels. This indicates also less extreme θ values, where the score transformations are the least precise and would more negatively affect the NRMSE. Second, it needs to be mentioned that simulation settings with small θ dispersion and low correlation between scales challenged the data-generating procedure, especially for the scales with *I* = 20 items. Although the data generation with $\sigma_{\theta }=0.5$ produced increasing factor correlations, their values were above the expected input values. Appendix Table C.1 to Table C.3 (see Appendix C in the Supplemental Online Materials) show the generated mean factor correlations. In any case, a higher similarity across scales increases the precision of the score equating. However, the advantage in the precision of the small θ dispersion is not as salient and close to what is found with $\sigma_{\theta }=1$ at same level of simulated similarity.

Based on Figures 8 and 9, the difficulty shifts between scales did not impact the transformation precision noticeably (see also Appendix C in the Supplemental Online Materials, Table C.8 to Table C.11). What matters most to the quality of the equating is that scales, measuring a similar construct, encompass the ability spectrum of the test-taker. Figures 8 and 9 illustrate the precision of the score transformation on the score continuum and contrast the (gain of) precision of the true-score equating with an identity equating. The x-axis of Figures 8 and 9 presents the range of possible raw scores $R_{X}$ for Scale X and $R_{Y}$ for Scale Y respectively. At a given raw score level on the x-axis, the y-axis values show the mean score differences and their confidence interval for the transformed and the observed score (Figure 8: $E({\hat{R}}_{Y}-R_{Y})$ , Figure 9: $E({\hat{R}}_{X}-R_{X})$ ). As the pattern repeats, only the figures with $\sigma_{\delta_{X}}=\sigma_{\delta_{Y}}=1$ and $\sigma_{\theta }=2$ for N=500 and I=10 are shown for the transformation from Scale X to Scale Y (Figure 8) and reversely (Figure 9). Ideally, with both methods working perfectly, both lines would be horizontal with mean differences between transformed and observed scores equal to zero. The confidence bands on the figures are set at one standard deviation above and below the mean. From left to right, the figures differ in the mean difficulty shift $\Delta_{\mathrm{XY}}=0$ , $\Delta_{\mathrm{XY}}=1$ , $\Delta_{\mathrm{XY}}=2$ , from top to bottom in size of input factor correlation, $\rho_{\mathrm{sim}}=0.25$ , $\rho_{\mathrm{sim}}=0.5$ , $\rho_{\mathrm{sim}}=0.75$ , and $\rho_{\mathrm{sim}}=1$ . Each plot also indicates the overall mean NRMSE of the true score and the identity equating for the repetitions in the specific setting. The identity equating is never more precise than the true-score equating approach. In general, for both approaches, the precision of the raw score transformation decreases toward the extremes of the scale. However, Figures 8 and 9 show that the size of the discrepancy between the two equating approaches is driven mainly by the difference in difficulty between the scales. The true-score equating approach is adjusting for the shift in difficulty so that the transformation precision is not affected by $\Delta_{\mathrm{XY}}$ up to 2 logits, also seen in Figures 8 and 9. In the present context, using the identity equating approach seems justifiable only with scales of similar difficulty, the loss of precision being minor. Also, with low scale similarity and no difficulty shift, the true-score and identity equating have similar performance. It is worthwhile mentioning, that for the left column, that is, in absence of shift, the plots of Figures 8 and 9 are symmetrical. With increasing shift, the symmetry assumption does not hold. The differences in shape with increasing shift, can be explained by the direction of the transformation. For example, when transforming very small scores from Scale X to Scale Y, from an easier to a more difficult scale, the transformation is limited by the lower range of Scale Y. This will not happen, when transforming from the difficult Scale Y to the easier scale.

## Empirical Example

This example briefly illustrates how insights gained from the simulation study can be helpful for empirical studies where true-score equating with a common person design is conducted. This example will show how the statistical characteristics, especially the psychometric properties, of two test forms can enable researchers to appropriately anticipate the equating precision prior to the equating.

### Data, Items, and Sampling

The data were kindly made available by the Asia Foundation, who conducted the World Health Organization’s and World Bank’s MDS in Afghanistan in 2019 in about 14,000 households (Sabariego et al., 2022; Shinwar et al., 2020). The MDS is generally used to collect information about health, functioning and environmental factors in the general population. Items from two MDS Modules were used for this example. One set of items is measuring health from a so-called capacity perspective (Module 5000) and the other set of items is measuring health from a so-called performance perspective (Module 4000). The items for the capacity metric ask about difficulties in daily life because of health problems. The items for the performance metric ask about the extent of problems the person experiences in daily life when also taking into account environmental factors that can be facilitating or hindering. Examples of environmental factors could be the attitudes and support of others, characteristics of the built environment (e.g., ramps), the availability of assistive devices (e.g., crutches) and home adaptations (e.g., wide doorways; World Health Organization, 2001). The response options indicate either increasing levels of difficulty (for the capacity) or increasing levels of problems (for the performance). For this example, the responses were recoded to represent three options—from no or mild difficulties/problems to extreme difficulties/problems—to ensure ordered thresholds.

Two scales of the same length were created for the purpose of this example (Table 2). The Performance Scale items of the MDS can be grouped into 16 functioning domains with one or more items per domain. For this example, the Performance Scale used the first item from each of the 16 functioning domains and 16 items from the MDS Capacity Scale that match the 16 domains of functioning assessed by the Performance Scale. The Capacity Scale presented two highly correlating items; the item assessing hearing and the item assessing communication. For simplicity, the item assessing hearing was removed so that the final Capacity and Performance Scales each counted 15 items respectively.

**Table 2.** Capacity and Performance Scale Item.

Domain	Capacity	Performance
Questionnaire Instruction	I have asked you many questions about kinds of problems you experience in your life. The next questions ask about difficulties you may have doing certain activities only because of your *health*. Please think about the last 30 days taking both good and bad days into account. Now thinking only about your health I want you to answer these questions *without* taking into account any help. Following questions on a scale from no difficulty to extreme difficulty.	In this module, I want to understand the kinds of problems you experience in your life. By problems I mean not getting things done in the way you want to or not getting them done at all. These problems may arise because of your health or because of the environment in which you live. They may arise because of the attitudes or behaviors of people around you. Please think about the last 30 days, taking both good and bad days into account. For each question, please tell me how much of a problem it was for you from no problem and to extreme problem.
Mobility	Do you have difficulty walking or climbing steps?	How much of a problem is standing up from sitting down for you?
Hand and Arm Use	Because of your health how much difficulty do you have doing things that require the use of your hands and fingers such as picking up small objects or opening a container?	How much of a problem is doing things that require the use of your hands and fingers such as picking up small objects or opening a container?
Self-Care	Because of your health how much difficulty do you have toileting?	How much of a problem is being clean and dressed?
Seeing	Do you have difficulty seeing even if wearing glasses?	How much of a problem do you have with seeing at a distance?
Pain	How much bodily aches or pains do you have?	How much of a problem is having pain in your day-to-day life for you?
Energy and Drive	How much difficulty do you have sleeping because of your health?	How much of a problem do you have with sleep?
Breathing	How much difficulty do you have with shortness of breath because of your health?	How much of a problem do you have with shortness of breath?
Affect	To what extent do you feel sad low or depressed because of your health?	How much of a problem do you have with feeling sad low or depressed?
Interpersonal Relationships	Because of your health how much difficulty do you have getting along with people who are close to you including your family and friends?	How much of a problem is getting along with people who are close to you including your family and friends?
Handling stress	Because of your health how much difficulty do you have coping with all the things you have to do?	How much of a problem is handling stress such as controlling the important things in your life?
Communication	Using your usual language, do you have difficulty communicating, for example understanding or being understood?	How much of a problem do you have with being understood using your usual language?
Cognition	Do you have difficulty remembering or concentrating?	How much of a problem is forgetfulness for you?
Household Tasks	How much difficulty do you have doing household tasks because of your health?	How much of a problem do you have with getting your household tasks done?
Community and Citizenship Participation	Because of your health how much difficulty do you have with joining community activities such as festivities religious or other activities?	How much of a problem do you have with doing things for relaxation or pleasure?
Caring for Others	How much difficulty do you have providing care or support for others because of your health?	How much of a problem do you have providing care or support for others?

**Figure 10.**
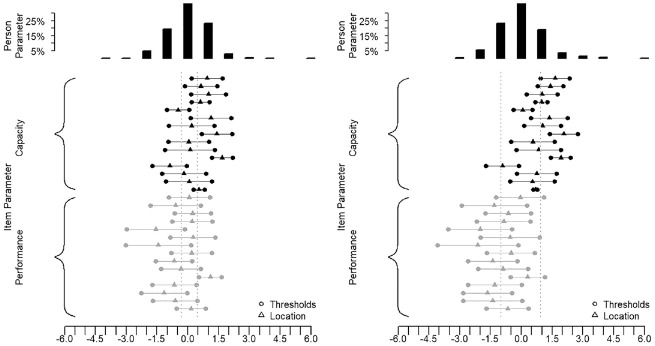
Targeting of Group 1 and Group 2.

For this illustrative example, we have selected two different groups of approximately *N* = 1,000 MDS participants. The first group was intentionally selected from the middle score range of the Capacity Scale and with scores covering the entire measurement continuum. In this first group, there was only a slight difference between the mean Capacity and the mean Performance scores for all participants (Figure 10). The second group, however, was selected to show a larger difference between the Capacity and the Performance mean scores, indicating that the participants in the second group had a more enabling environment, such as more social support, better living conditions, better infrastructures, or assistive devices.

### Analysis Design

This example runs, for each group, through all the steps described in the simulation study to obtain equated scales by means of true-score equating in a common person design. It is expected that, if the separate analyses in Step A support good fit, the factor correlation ( $\rho_{\mathrm{XY}}$ ) from the 2-dimensional PCM and the information on targeting from the concurrent PCM analysis will allow to anticipate the quality of the resulting score transformations for each group. The transformation precision (NRMSE) is then discussed, given the scales and population characteristics.

### Results

**Figure 11.**
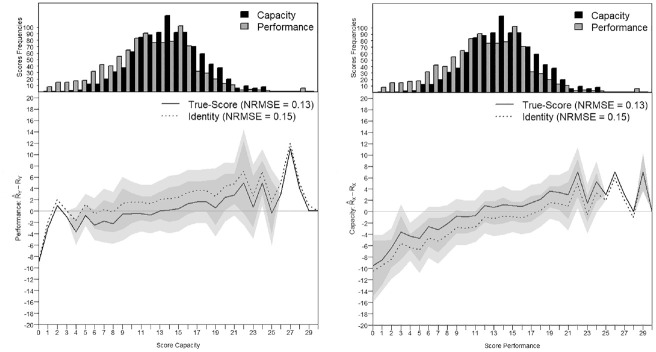
Score Transformation Precision From the Capacity to the Performance Metric and Reversely in Group 1. *Note.* NRMSE = normative root mean square error.

**Figure 12.**
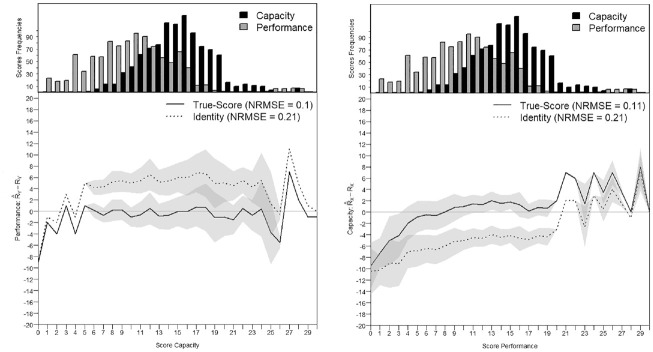
Score Transformation Precision From the Capacity to the Performance Metric and Reversely in Group 2. *Note.* NRMSE = normative root mean square error.

Table 3 presents the fit statistics for the Capacity and the Performance Scales, once with the group of participants selected from the middle score range and once with the group of participants with a lower Performance score, indicating higher functioning through a more enabling environment. The results of the analysis are interpreted as described in the PCM method section in Appendix A in the Supplemental Online Materials. Table 3 shows that for both groups the separate and anchored analyses of the scales yield items with ordered thresholds, good fit, and unidimensionality. The highest difference between observed and mean residual item correlation was 0.24, indicating a light dependency between a pair of items. In fact, the item about *toileting* and the item about *hand use* from the Capacity Scale had a higher residual correlation with r=0.17 after anchoring. A residual correlation of r=0.17 was also found in the Performance Scale between the items on coping and pain. Figure 10 shows the distribution of the item and person parameters. In the second group, the mean difficulty of the Performance Scale is shifted toward the right because, in this example, the second sample was drawn intentionally with individuals having more difficulty in terms of their capacity compared with their performance in daily life. The dotted lines indicate the mean scores for each scale. Figure 10 shows that the shift in difficulty is bigger in the second group, that is, $\Delta_{\mathrm{XY}}=$ 1.25 logits. But in the first group, the shift is also not zero and equals $\Delta_{\mathrm{XY}}=$ 0.5 because many persons have some facilitating effect from their environment. The person parameters are centered to equal zero, which is the default parameterization in the R package *mirt*. Similar to Figures 8 and 9, Figures 11 and 12 show the precision of the score transformation on the score continuum for each group, either after true-score equating or when using a simple identity equating. Figures 11 and 12 also show both the NRMSE values for the true-score and the identity equating and confirm the higher precision of score transformation when applying true-score equating especially in the presence of a higher difficulty shift. In Figure 12, transforming the Capacity Scores to conform to the Performance metric in group 2 results in NRMSE=0.11 after true-score equating and NRMSE=0.24 after identity equating. The two Figures also show that the departures from the horizontal line with transformation error = 0 increase in the extremes especially when the frequency of observable scores is low, as seen in the person parameters distribution at the top.

**Table 3.** Results by Step for the True-Score Equating.

Analysis Step and Measurement Assumption Tested	Group 1		Group 2	
	Capacity	Performance	Capacity	Performance
Two-dimensional PCM				
Factor correlation—ρ	0.78		0.92	
A) Separate analyses				
Item fit	Good	Good	Good	Good
Thresholds	Ordered	Ordered	Ordered	Ordered
LID	0.24	0.23	0.21	0.2
Dimensionality (%sig. *T*-tests)	1.29%	3.36%	3.18%	2.12%
Targeting				
Item parameters—μ_δ_(σ_δ_)	0.18(0.4)	0.74(0.52)	–0.09(0.39)	1.28(0.51)
Person parameters—μ_θ_(σ_θ_)	0(0.89)	−0.01(1.21)	0(0.9)	0(1.4)
B) Concurrent analyses				
Dimensionality (%sig. *T*-tests)	7.22%		5.58%	
Item parameters—μ_δ_(σ_δ_)	0.18(0.42)	0.68(0.48)	−0.1(0.42)	1.15(0.45)
Person parameters—μ_θ_(σ_θ_)	0(0.9)		0.01(1.05)	
C) Anchored analyses				
Item fit	Good	Good	Good	Good
Thresholds	Ordered	Ordered	Ordered	Ordered
LID	0.23	0.23	0.21	0.21
Dimensionality (%sig. *T*-tests)	1.38%	2.37%	3.27%	1.64%
Targeting				
Item parameters—μ_δ_(σ_δ_)	0.18(0.42)	0.68(0.48)	−0.1(0.42)	1.15(0.45)
Person parameters—μ_θ_(σ_θ_)	0(0.9)	−0.02(1.15)	0(0.95)	−0.03(1.33)

*Note.* PCM = partial credit model; LID = Local Item Dependency: the highest residual correlation.

In the first group, the factor correlation was $\rho_{\mathrm{XY}}=$ 0.78, the dispersion of the person parameters in the concurrent calibration in Step B was equal to $\sigma_{\theta }=$ 0.9 and the mean difficulty and dispersion of the item parameters were $\mu_{\delta }=0.1$ and $\sigma_{\delta }=0.45$ for the Capacity Scale and $\mu_{\delta }=0.68$ and $\sigma_{\delta }=0.46$ for the Performance Scale. The closest simulation setting, to what is found in the empirical data for Group 1, was the simulated setting with $\rho_{\mathrm{sim}}=0.75$ , $\sigma_{\theta }=1$ , $\mu_{\delta }=0$ for both scales. The lowest simulated item dispersion was $\sigma_{\delta }=1$ , which is higher than what is found in the empirical data. The observed difficulty shift between the two scales was $\Delta_{\mathrm{XY}}=$ 0.5, which is between $\Delta_{\mathrm{XY}}=0$ and $\Delta_{\mathrm{XY}}=1$ . The simulation had shown that increasing the $\Delta_{\mathrm{XY}}$ up to 2 logits had no effect on the precision of the scores transformations. In scales with ten items, the most similar simulation settings showed NRMSE values of 0.18 to 0.19 (see Appendix C in the Supplemental Online Materials, Table C.10). With 20 items, the NRMSE dropped to 0.16 to 0.17 (see Appendix C in the Supplemental Online Materials, Table C.11). For the empirical data of group 1, the observed NRMSE was 0.13, from the Capacity to the Performance Scale score and reversely. This observed transformation precision for the empirical data is somewhat better than what we would expect based on the simulation outcomes. However, the empirical data may have benefited from the lack of extreme values, which would have impacted the NRMSE more negatively. Cases with very high performance problems or very high difficulties in terms of their capacity were underrepresented (see Figures 11 and 12) compared with the simulated data.

In the second group, the factor correlation was $\rho_{\mathrm{XY}}=$ 0.92, in the concurrent calibration the dispersion of the person parameters was equal to $\sigma_{\theta }=$ 1.05, the mean difficulty and dispersion of the item parameters were $\mu_{\delta }=-0.17$ and $\sigma_{\delta }=0.41$ for the Capacity Scale and $\mu_{\delta }=1.24$ and $\sigma_{\delta }=0.45$ for the Performance Scale. The observed difficulty shift between the Capacity and the Performance scale was $\Delta_{\mathrm{XY}}=$ 1.25. The closest simulated settings had a similarity of $\rho_{\mathrm{sim}}=1$ , a $\sigma_{\theta }=1$ , difficulty shifts between $\Delta_{\mathrm{XY}}=1$ or $\Delta_{\mathrm{XY}}=2$ , and a dispersion for the item parameters $\sigma_{\delta }=1$ . In scales with 10 items, the closest simulation setting showed NRMSE values of 0.13 to 0.14 (see Appendix C in the Supplemental Online Materials, Table C.10). With 20 items, the NRMSE dropped to 0.09 to 0.1 (see Appendix C in the Supplemental Online Materials, Table C.11). For the empirical data of group 2, the observed NRMSE was 0.1 from the Capacity to the Performance Scale score and 0.11 for the reverse transformation. For identity equating, the NRMSE was 0.21. The empirical example also supports the robustness of true-score equating, but not identity equating, in the presence of shifts in difficulty between the scales to be equated found in the simulation study. Thus, the transformation precision that can be expected based on the psychometric characteristics found in the second group agrees with the findings in the simulation study.

## Discussion

This simulation study supports that, when equating scales, construct similarity is essential for a good score transformation precision. The factor correlation between scales, in other words, their construct similarity, explained to a large extent the score transformation quality. Higher correlation goes along with better transformation precision. The importance of the scale similarity in equating is not surprising, but this study also shows the relevance of the targeting, something few other studies have investigated so far. The precision of the score transformations improves with good targeting, when the scales have a broad measurement scope that embraces the ability spectrum of the test-taker. With highest similarity, the quality of the targeting did not have any notable impact on the transformation quality. With lower similarity of the scales, however, the impact of insufficient targeting was reinforced and further deteriorated the transformation precision. For a given θ dispersion, transformation from a scale with smaller δ dispersion toward a scale with larger δ dispersion was always more precise. This simulation study also showed that, when regarded separately from targeting, shifts in difficulty between scales have a negligible effect for up to 2 logits difficulty differences.

With diminishing correlation between dimensions, the equated scales become more dissimilar, negatively affecting the score transformation precision. Hart et al. (2006) remarked that strict unidimensionality and local independence are difficult to achieve in practice and that generally scale developers aim for “essential unidimensionality” (Hays et al., 2000). Studies interested in testing the effect of the degree of similarity between scales in scale equating, especially under various equating strategies, can be found in the literature (Bolt, 1999; Brossman & Lee, 2013; Hanson & Beguin, 2002). Studies investigating effects of within-scale dimensionality on equating are also found (Kim et al., 2019; Yen, 1984). Our study only focused on the similarity of the two scales to be equated, that is, the between-scale dimensionality.

When observing equating in practice, similarity of the scales is often an implicit assumption that transfers into practice as a subjective evaluation of what the scales to equate are aiming to assess. Unidimensionality, or sufficient unidimensionality for a robust score transformation is not sufficiently supported by only assuming that scales measure a common latent trait. As mentioned in the introduction, also characteristics of the assessment, such as for example the response options or the general phrasing of the scale items, can strongly affect the degree of similarity of scales.

In practice, the validity of a transformation rule between equated scales is often assumed if the single and equated scales fit the Rasch model but is rarely discussed in terms of transformation precision. We recommend always testing the between-scale dimensionality, for example by means of multidimensional Rasch analysis, which provides a tangible value of the degree of association between two scales. Good psychometric properties of the scales to be equated are not a sufficient guarantee for good score transformation precision. The separate analyses of the scales in this simulation presented generally acceptable to good psychometric properties. But, despite otherwise good psychometric properties, already with factor correlations of 0.75, the quality of a score transformation is negatively impacted, with average score departures up to 25% points of the total scale range. When scales have been equated through true-score equating in a common person design, we recommend also reporting a statistic on the degree of similarity of the equated scales and a statistic on the observed-score transformation precision to have two objective measures on the general quality of the equating. This study used the factor correlation between scales issued by a two-dimensional PCM analysis to determine the degree of similarity of scales. Given different scale lengths, score transformation precision was summarized by means of the NRMSE, but other statistical coefficients are possible (Kolen & Brennan, 2014).

Studies reporting the degree of associations are rare, and critical cut-off values for judging the degree of association are missing with no consensus on what is sufficient unidimensionality (Martin et al., 2007). The empirical example showed that the precision of score transformations can be anticipated to some degree by knowing the targeting and similarity of the scales to equate. The NRSME values found in the simulation can be expected to provide estimates on the degree of departure in real data contexts with scales presenting similar psychometric properties as those simulated in the study. The loss of precision that researcher or clinicians are willing to accept also depends very much on the purpose of the assessment. More precise equating would be expected for clinical measurement at individual level than for measurement at population level, for example.

When the unidimensionality between the equated scales does not hold, alternative strategies that could be applied in practice are only marginally discussed in the literature. For example, multidimensional equating procedures exist (Hirsch, 1989; R. D. Gibbons et al., 2014). Also, in the presence of multidimensionality, testlet approaches are often applied. In testlet-based equating, also called score co-calibration, the raw scores of the scales are submitted to a concurrent calibration instead of the items (Surla, 2020). Unidimensional true-score equating was applied here, as the method is commonly applied in practice and probably more accessible when equating with the Rasch model in a common person design. It would be worthwhile to compare the score transformation precision of these alternative procedures with the unidimensional true-score, item-based equating approaches when the construct similarity is challenged.

In the research literature, the effects of targeting are more widely studied in terms of differences in difficulty across equated scales than in terms of differences in the degree of dispersion of the θ versus the δ parameter. This study also showed that targeting moderates the equating precision. The negative effect of low similarity is reinforced with poorly targeted scales, while with high scale similarity, the effect of poor targeting is marginal. This is an important finding. Studies that account for the quality of the θ and δ overlap are barely found. However, scales with insufficient targeting are commonly observed in the practice. The development of comprehensive but parsimonious scales that are able to assess individuals at all levels of ability are a challenge. Skewed distributions of θ parameter, floor or ceiling effects are commonly observed in health research, where the extremes of scales that measure the very worst health outcome are often sparsely populated. The findings of this simulation study support the relevance of targeting in score equating and its moderating effect in face of scale similarity. This simulation study, further, confirms that differences in the difficulty of the two scales per se have only a small effect on the quality of the equating. This goes along with findings in methodological research related to this topic, for example, with regard to the drift of dichotomous items under the 2PL (Wells et al., 2002) or under the Rasch model with non-normal parameters distributions (Witt et al., 2003), or across IRT models (Rupp & Zumbo, 2003) that reported a negligible effect of shift on resulting θ estimates. All these studies used parameters from real assessments in their simulation. The size of the difficulty shift or drift in Witt et al. (2003) ranged from −0.5 to +0.5 logits and was at most 0.8 logits in Rupp and Zumbo (2003). This study confirms the robustness of the Rasch-based equating in face of item difficulty shifts up to two logits.

Drawing strong conclusions based on this simulation with a limited number of settings should be considered with caution, as for any simulation study. Only some assessment characteristics have been varied and observed systematically within a limited range. The sample sizes used in this study are representative for the health research field where equating studies are the most commonly undertaken. However, health assessment can also take place as part of national or international surveys in the general or more specific populations providing larger health data pools. The number of response options and the discrimination of items were kept constant to avoid disordering of thresholds and item misfit, as these were out of the research scope. More than three response options could have been considered. Measurement scales with more than five response options are not uncommon and are known to result in a more refined assessment (Preston & Colman, 2000). To be fair, more response options typically also go along with response threshold ordering issues. In that sense, this simulation only used well-behaved items, producing an uncommonly optimal equating situation. Threshold disordering and item misfit, in practice, would require undertaking actions to improve the metric and its compliance to the Rasch model, for example, through deletion of misfitting items and collapsing of the disordered response categories (Salzberger, 2015; Tennant & Conaghan, 2007). In an extensive simulation, addressing and solving these issues individually for each simulated equating setting was not feasible. Many more aspects that are relevant in applied psychometrics could be varied and would have further increased the generalizability of this simulation study. For example, locally dependent items, items with differential item functioning, within-scale multidimensionality, unequal score ranges across scales, or even smaller sample sizes, can all be expected to moderate the score transformation precision across equated scales and could be investigated in future studies.

This simulation study has also several strengths. Many methodological equating studies use existing data or simulate data that are conditioned on parameters derived from analyses with the existing data instead of generating data following a simulation design. Real data or simulations conditioned on actual parameters describe realistic data collection settings. However, the generalizability of findings of such studies may be diminished and confined to settings with similar characteristics. Artificial data generate and target effects precisely through a standardized setting. Through the R environment (R Core Team, 2020), this study could automatize the simulation of data controlling for the degree of similarity between scales and the characteristics of the distributions of the δ and θ . Furthermore, most simulation studies that investigate equating with the Rasch or IRT models use dichotomous data. This preference can be explained by the extensive equating research undertaken in the educational field. Educational scales typically provide so-called number-correct scores that are derived from the dichotomous correct/incorrect ratings of items from educational tests. However, in psychological or health measurement, scales using a polytomous response type are largely preferred to express, for example, the degrees to which one agrees with questionnaire items.

## Conclusion

This study showed that when observing similarity, equity and targeting of scales, the similarity was the most important determinant of equating precision. Similarity indicates that scales measure a common construct. The targeting of the scales also affected the precision of the equating, with a moderating effect and increasing impact when the similarity of the scales decreased. Differences in difficulty across scales had no notable effect on the precision of score transformations across equated scales for true-score equating. In that sense, the requirement for equity in true-score equating may not be a strong requirement when difficulty shifts are below two logits. This simulation study highlights the effects of scale characteristics and departures from similarity and suggests ways to improve the quality and transparency of the equating procedure, such as the assessment of the factor correlation by means of the multidimensional PCM and an analysis of the transformation precision. According to Andrich (1988), who stated that at some level of precision any construct is undimensional, we sense a danger in a purely subjective or qualitative evaluation of the similarity of constructs to be equated. Beyond fit statistics, equating studies using the true-score equating approach would benefit from reporting the dimensionality of the concurrent calibration of scales and questioning the similarity of the equated scales objectively. Based on the findings of the simulation study and the empirical example, it can be expected that information on the distribution of person and item parameters, and the similarity of the two scales, would allow to appropriately anticipate the equating precision prior to the equating.

## Supplemental Material

sj-pdf-1-epm-10.1177_00131644221143051 – Supplemental material for What Affects the Quality of Score Transformations? Click here for additional data file. Supplemental material, sj-pdf-1-epm-10.1177_00131644221143051 for What Affects the Quality of Score Transformations? by Carolina Fellinghauer, Rudolf Debelak and Carolin Strobl in Educational and Psychological Measurement
