# Supplementary material for: What Affects the Quality of Score Transformations? Potential Issues in True-Score Equating Using the Partial Credit Model
Source: Educ Psychol Meas. 2023 Jan 13;83(6):1249–90. doi: 10.1177/00131644221143051 (PMC10638984; doi:10.1177/00131644221143051)
Supplement: sj-pdf-1-epm-10.1177_00131644221143051 – Supplemental material for What Affects the Quality of Score Transformations? [file sj-pdf-1-epm-10.1177_00131644221143051.pdf]

Appendix A

Partial Credit Model Analysis

In practice, for a Rasch analysis, here with the partial credit model (PCM), a series of measurement assumptions are tested, and the fit of the data to the model is determined with fit indices. Typically, a Rasch analysis checks whether the data collected through an assessment form with ordinally scaled items adhere to specific characteristics essential for measurement. In practice, the measurement assumptions tested are stochastic ordering, monotonicity, the absence of local item dependencies (LID), unidimensionality, and the absence of differential item functioning (DIF) (Tennant and Conaghan 2007). If these assumptions are met, a test form is 'fit' for measurement and delivers an interval scaled score. For this study, at each step of the equating process, whether the data adhered to the measurement assumptions was investigated and relevant fit indices of the PCM were reported as supplementary material in Appendix B.

Stochastic ordering is supported when responses to test items are a function of the abilities of the test-taker and the item difficulties. If the items fit the PCM, the mean squared residuals (MSQ) values per item are expected close to one; this is also called the Outfit. The Infit is a weighted total statistic for the MSQ (R. M. Smith, Schumacker, and Bush 1998). In this simulation study, items showing Infit and Outfit statistics between 0.5 and 1.5 were considered 'productive for measurement' (Linacre 2002).

Good targeting of a test form indicates that the difficulty of an assessment matches the ability level of the test-taker. In general, targeting requires that the mean of the item parameter (IP) and the mean of the person parameter (PP) distribution correspond, supporting that the test form is neither too difficult nor too easy for the test-taker. In this study, the targeting of the test forms was manipulated by design. To challenge the equity requirement of the equating, the mean IP of the forms was shifted away from zero but the PP were kept centered. Also, the measurement scope was varied by increasing the item dispersion targeted on simulated population of test-takers with increasing dispersion of ability; see the Simulation Study section for details.

Monotonicity implies strictly increasing difficulty estimates for the item response thresholds. In practice, disordered response thresholds in some items are more the rule than the exception. Especially, items with many response options and poorly discriminating middle categories tend to show breaches to the threshold ordering (Andrich and Marais 2019; Andrich and Luo 2003). The present study simulated polytomous items with three response categories.

LID often occurs when items are redundant and measure approximately the same or very similar aspects of a latent construct, e.g., SF-36 items: "walking 100 yards", "walking half a mile", and "walking more than a mile" in Horton and Tennant (2011). Typically, the correlations of the standardized Rasch residuals, or Q_3-values, are used to detect LID (Yen 1984). High positive correlations indicate LID. Negative residual correlation can reveal multidimensionality of the form. LID leads to inflated reliability estimates (Baghaei 2008). Marais (2013) recommends evaluating LID relative to the average residual correlations, as the magnitude of the residual correlations depends on the number of items. Christensen, Makransky, and Horton (2017) formalized this, suggesting that if the largest Q_3 value is more than 0.2 above the average, i.e., if $Q_3^{\star} = Q_{3,max} - \bar{Q_3} > 0.2$ this indicates departure from independence.

For a valid raw score, a test form should measure only one latent construct. With a test form assessing data over several dimensions, the calculation of one unique total score is not valid anymore. Principal component analysis (PCA) of the standardized Rasch residuals tests unidimensionality by searching for non-random patterns in the analysis residuals (E. V. J. Smith 2002). If unidimensionality holds, the residuals are free of any salient component loading structure. Unidimensionality can be tested by mean of pairwise t-tests that compare the PP estimates ($\hat{\theta}$) from separate but anchored Rasch analyses, with items that loaded positively (PC^{+}) or negatively (PC^{-}) on the first component of the PCA of a common calibration. The anchoring of the separate analyses uses the IP estimates

of a common calibration of the items to be tested for unidimensionality. The t-test involves each individual pair of $\hat{\theta}$ estimates, here $\hat{\theta}_{PC+}$ and $\hat{\theta}_{PC+}$ and their respective measurement error ($SE_{\hat{\theta}_{PC+}}$ and $SE_{\hat{\theta}_{PC-}}$) using the following formula:

$$t = \frac{\hat{\theta}_{PC+} - \hat{\theta}_{PC-}}{\sqrt{SE^2_{\hat{\theta}_{PC+}} + SE^2_{\hat{\theta}_{PC-}}}}.$$

The percentage of significant t-tests should not exceed 5% (Andrich and Marais 2019). This simulation intentionally varies degrees of similarity between test forms but expects within test form unidimensionality, the t-tests and Q_3 values are expected to reflect this.

When reporting psychometric analyses, the reliability coefficient is often considered a critical statistic to support the quality of an assessment scale (Colton et al. 1997). In practice, when deciding to include an assessment instrument in a study or a survey, the information about its reliability is central. In modern test theory, reliability uses the derived PP (θ) to describe how well an assessment instrument is expected to differentiate between levels of ability among test taker. The reliability estimate entirely relies on the distribution of the $\hat{\theta}$ estimates, specifically their variance $\sigma^2_{\hat{\theta}}$, and the mean of the measurement error ($MSE_{\hat{\theta}}$). The R package `mirt` uses an empirical estimate of the reliability formalized as

$$\hat{\rho}^2_{\theta\hat{\theta}} = 1 - \frac{MSE_{\hat{\theta}}}{\sigma^2_{\hat{\theta}} + MSE_{\hat{\theta}}} = \frac{\sigma^2_{\hat{\theta}}}{\sigma^2_{\hat{\theta}} + MSE_{\hat{\theta}}}.$$

This reliability coefficient indicates how much the measurement process has reduced the uncertainty of the PP estimates (Mislevy et al. 1992). The empirical reliability coefficient is an alternative form of the Person Separation Reliability (PSR) that is found in Rasch software (Linacre 2015; Andrich, Sheridan, and Luo 2010) and R packages (Mueller 2020; Mair, Hatzinger, and Maier 2021). Typically, a PSR of 0.8 or above is interpreted as indicating good reliability. Excellent reliability can be expected with PSR values above 0.9 (Tennant and Conaghan 2007). The simulation indirectly affects the test forms' reliability by varying the dispersion of the person estimates.

The PP of this study were estimated with the weighted likelihood estimation (WLE) approach of Warm (Warm 1989). WLE reduces bias by decreasing the weight of inconsistent items, i.e., items with large residuals, to a considerable degree (Schuster and Yuan 2011) and further provides finite estimates for the extreme score (Warm 1989). For Rasch models, such as the PCM (Masters 1982), all test-takers with the same raw score (R) have the same PP estimate ($\hat{\theta}$), independently of the response pattern.

Finally, a complete description of the measurement assumptions tested in an analysis with a Rasch model would also mention the analysis of differential item functioning (DIF) that examines whether each item is free of subgroup effects, e.g., gender or age effects. No DIF analysis was undertaken in this study as all items were simulated free of DIF.

References

Andrich, D., and G. Luo. 2003. "Conditional Pairwise Estimation in the Rasch Model for Ordered Response Categories Using Principal Components." *Journal of Applied Measurement* 4: 205–21.

Andrich, D., and I. Marais. 2019. *A Course in Rasch Measurement Theory: Measuring in the Educational, Social and Health Sciences*. Singapore: Springer.

Andrich, D., B. Sheridan, and G. Luo. 2010. "Rasch Models for Measurement: Rumm2030." Perth, Western Australia: RUMM Laboratory Pty Ltd.

Baghaei, P. 2008. "Local Dependency and Rasch Measures." *Rasch Measurement Transactions* 21 (3): 1105–6. https://www.rasch.org/rmt/rmt213b.htm.

Christensen, K. B., G. Makransky, and M. Horton. 2017. "Critical Values for Yen's Q3: Identification of Local Dependence in the Rasch Model Using Residual Correlations." *Applied Psychological Measurement* 41 (3): 178–94. https://doi.org/10.1177/0146621616677520.

Colton, D. A., X. Gao, D. J. Harris, M. J. Kolen, D. Martinovich-Barhite, T. Wang, and C. J. Welch. 1997. "Reliability Issues With Performance Assessments: A Collection of Papers." *ACT Research Report Series* 3: 1–134. https://www.act.org/content/dam/act/unsecured/documents/ACT_RR97-03.pdf.

Horton, M., and A. Tennant. 2011. "Applying Rasch Analysis to the SF-36 Physical Function Scale: Effect of Dependent Items." *Trials* 12 (1): A75. https://doi.org/10.1186/1745-6215-12-S1-A75.

Linacre, J. M. 2002. "What Do Infit and Outfit, Means-Square and Standardized Mean?" *Rasch Measurement Transactions* 16 (2): 878. https://www.rasch.org/rmt/rmt162f.htm.

———. 2015. "Winsteps Rasch Measurement Computer Program User's Guide." Edited by O. R. Beaverton. Winsteps.com.

Mair, P., R. Hatzinger, and M. J. Maier. 2021. *eRm: Extended Rasch Modeling*. https://cran.r-project.org/package=eRm.

Marais, I. 2013. "Local Dependence." In *Rasch Models in Health*, 111–30. John Wiley & Sons, Ltd. https://doi.org/10.1002/9781118574454.ch7.

Masters, G. N. 1982. "A Rasch Model for Partial Credit Scoring." *Psychometrika* 47: 149–74. https://doi.org/10.1007/BF02296272.

Mislevy, R. J., A. E. Beaton, B. Kaplan, and K. M. Sheehan. 1992. "Estimating Population Characteristics from Sparse Matrix Samples of Item Responses." *Journal of Educational Measurement* 29 (2): 133–61.

Mueller, M. 2020. *Iarm: Item Analysis in Rasch Models*. https://CRAN.R-project.org/package=iarm.

Schuster, C., and K.-H. Yuan. 2011. "Robust Estimation of Latent Ability in Item Response Models." *Journal of Educational and Behavioral Statistics* 36 (6): 720–35. https://doi.org/10.3102/1076998610396890.

Smith, E. V. Jr. 2002. "Detecting and Evaluating the Impact of Multidimensionality Using Item Fit Statistics and Principal Component Analysis of Residuals." *Journal of Applied Measurement* 3 (2): 205–31.

Smith, R. M., R. E. Schumacker, and M. J. Bush. 1998. "Using Item Mean Squares to Evaluate Fit to the Rasch Model." *Journal of Outcome Measurement* 2 (1): 66–78.

Tennant, A., and P. G. Conaghan. 2007. "The Rasch Measurement Model in Rheumatology: What Is It and Why Use It? When Should It Be Applied, and What Should One Look for in a Rasch Paper?" *Arthritis Care & Research* 57 (8): 1358–62. https://doi.org/10.1002/art.23108.

Warm, T. A. 1989. "Weighted Likelihood Estimation of Ability in Item Response Theory." *Psychometrika* 54 (3): 427–50. https://doi.org/10.1007/BF02294627.

Yen, W. M. 1984. "Effects of Local Item Dependence on the Fit and Equating Performance of the Three-Parameter Logistic Model." *Applied Psychological Measurement* 8 (2): 125–45. https://doi.org/10.1177/014662168400800201.

Appendix B

```
library(foreach)
library(doParallel)
library(readr)



#The settings were run separately and aggregated afterwards
specific <- "_I10_N500"  # "_I10_1000", "_I20_N500", "I20_N1000"
#


#
# #Steps


# # A. Parameter Setup ---------------------------------

sd_values0 <- c(0.5, 1, 2) #1, 2 evtl 0.5
cor_values <- c(0.25, 0.5, 0.75, 1) #0.5
n <- as.numeric(substring(strsplit(specific, "_")[[1]][3], 2))
nvar <- as.numeric(substring(strsplit(specific, "_")[[1]][2], 2))
idif <- c(0,1,2)
scope0 <- c(1,2)
simnumb <- 1:500

# Simulation runs through the matrix
Combis_Grid <- as.data.frame(expand.grid(cor_values, idif,
                  sd_values0, scope0, scope0, n, nvar,
                  equate, simnumb))
colnames(Combis_Grid) <- c("Cor", "idif", "SD",  "Scp1", "Scp2", "n", "nvar", "SIM")

# ..Combis_Grid ----------------------------------------

#Unequal item parameter setting, only in one direction

Combis_Grid <- Combis_Grid[-which(Combis_Grid$Scp1 == 2 & Combis_Grid$Scp2 == 1),]
rownames(Combis_Grid) <- 1:nrow(Combis_Grid)


# #Interpolation for potential missing values in conversion table

interpolna <- function(theta) { #x being a vector or column of theta in a conversion table
  na = which(is.na(theta))
  theta[na] = return(
```

```
    predict(
      interpSpline((0:(length(theta) - 1))[-na],
                   theta[-na]), (
                     0:(length(theta) - 1))[na])$y)
}


#..Analysis function

Equate_Sim <- function(x, Save_Thetas = TRUE, algoType = NULL) {

    set.seed(091075 + as.numeric(x["SIM"]))


    # ..algoType
    if(is.null(algoType)) {
      TypAlgo = "WLE"
    } else {
      TypAlgo = algoType
    }


  # ..Sigma & Shift

  Covariance <- x["Cor"] * x["SD"] * x["SD"]
  Sigma <- matrix(c(x["SD"]^2, Covariance, Covariance, x["SD"]^2), 2)
  Sigma <- apply(Sigma, 1, as.numeric)

  #set the shift

  if(x["idif"] == 0){Mu = c(0, 0)}
  if(x["idif"] == 1){Mu = c(-0.5, 0.5)}
  if(x["idif"] == 2){Mu = c(-1, 1)}

# ..2-DIM Rasch parameter fixing for the DGP

  if(x["nvar"] == 10){
    spec <- "
              F1 = 1-10
              F2 = 11-20
              START = (1-10, a1, 1.0)
              START = (11-20, a2, 1.0)
              FIXED = (1-10, a1)
              FIXED = (11-20, a2)
              FREE = (GROUP, COV11)
```

```
            FREE = (GROUP, COV22)
            COV = F1*F2 "
}

if(x["nvar"] == 20){
  spec <- "
            F1 = 1-20
            F2 = 21-40
            START = (1-20, a1, 1.0)
            START = (21-40, a2, 1.0)
            FIXED = (1-20, a1)
            FIXED = (21-40, a2)
            FREE = (GROUP, COV11)
            FREE = (GROUP, COV22)
            COV = F1*F2 "
}

# ..CombisGrid Row
x <- as.numeric(x)

names(x) <- c("Cor", "idif", "SD", "Scp1", "Scp2", "n", "nvar", "SIM")


##gpcm with always same constrained on slopes
a <- matrix(0, ncol = 2, nrow = x["nvar"] * 2)
a[1:x["nvar"], 1] <- rep(1, x["nvar"])
a[(1:x["nvar"]) + x["nvar"], 2] <- rep(1, x["nvar"])
colnames(a) <- c("a1", "a2")


if(x["Cor"] < 1) {

a1_Fx <- rnorm(x["nvar"], 0, x["Scp1"])
a2_Fx <- a1_Fx[order(a1_Fx)]
b1_Fx <- abs(rnorm(x["nvar"], 0.7, 0.15))
c1_Fx <- a2_Fx - b1_Fx
d1_Fx <- a2_Fx + b1_Fx
ok_Fx <- cbind(c1_Fx, d1_Fx)

a1_Fy <- rnorm(x["nvar"], 0, x["Scp2"])
a2_Fy <- a1_Fy[order(a1_Fy)]
b1_Fy <- abs(rnorm(x["nvar"], 0.7, 0.15))
c1_Fy <- a2_Fy - b1_Fy
d1_Fy <- a2_Fy + b1_Fy
ok_Fy <- cbind(c1_Fy, d1_Fy)
```

```
  ok <- rbind(ok_Fx, ok_Fy)
  }


  if(x["Cor"] == 1) {

    a1_Fx <- rnorm(x["nvar"], Mu[1], x["Scp1"])
    a2_Fx <- a1_Fx[order(a1_Fx)]
    b1_Fx <- abs(rnorm(x["nvar"], 0.7, 0.15))
    c1_Fx <- a2_Fx - b1_Fx
    d1_Fx <- a2_Fx + b1_Fx
    ok_Fx <- cbind(c1_Fx, d1_Fx)

    a1_Fy <- rnorm(x["nvar"], Mu[2], x["Scp2"])
    a2_Fy <- a1_Fy[order(a1_Fy)]
    b1_Fy <- abs(rnorm(x["nvar"], 0.7, 0.15))
    c1_Fy <- a2_Fy - b1_Fy
    d1_Fy <- a2_Fy + b1_Fy
    ok_Fy <- cbind(c1_Fy, d1_Fy)

    ok <- rbind(ok_Fx, ok_Fy)
    }

  dIRT <- cbind(rep(0, x["nvar"]), ok)
  d = dIRT
  d[, 3] <- -1 * (d[, 3] + d[, 2])
  d[, 2] <- -1 * d[, 2]
  colnames(d) <- NULL


x["dIRT_shift"] <- mean(apply(dIRT[, c(2, 3)], 1, mean))



  # B1) DGP-------------------------

if(x["Cor"] < 1) {
  repeat {
    dataset <- mirt::simdata(a,
                            d,
                            x["n"],
                            sigma = Sigma,
                            mu = rev(Mu),
                            itemtype = 'gpcm')
      if(
```

```
        (is.null(dim(apply(dataset, 2, table))) == FALSE | length(
          unlist(
            apply(dataset, 2, table))) > 0) & all(
              apply(dataset, 2, var) > 0)) {
    break
     }
  }
} else {
 repeat {
    dataset <- mirt::simdata(a = rep(1, x["nvar"] * 2),
                            d,
                            Theta = as.matrix(rnorm(x["n"],
                            0,
                            x["SD"])),
                            itemtype = 'gpcm')
      if(
        (is.null(dim(apply(dataset, 2, table))) == FALSE | length(
          unlist(
            apply(dataset, 2, table))) > 0) & all(
              apply(dataset, 2, var) > 0)) {
      break
      }
    }
}

Data_F1 <- dataset[, 1:x["nvar"]]
Data_F2 <- dataset[, (1:x["nvar"]) + x["nvar"]]

Rsum_F1 <- rowSums(Data_F1)
Rsum_F2 <- rowSums(Data_F2)



#  2-DIM Rasch ------------------------------------------------------


TOL <- 0
repeat {
TOL <- TOL + 0.005
mod_2dimensional <- tryCatch({mirt(cbind(Data_F1, Data_F2),
                    model = spec, itemtype = "gpcm",
                    SE = TRUE, TOL = TOL, verbose = FALSE)},
                    warning = function(w) {NULL})
if(length(mod_2dimensional) > 0) {
x["TOL"] <- TOL
```

```r
break
}
}

x["cor_all"] <- as.numeric(summary(mod_2dimensional)$fcor[1, 2])[1]


## C) Separate PCM---------------------------------
mod1_start <- mirt(Data_F1, 1, itemtype = "Rasch") #Items from Dim1, not anchored
mod2_start <- mirt(Data_F2, 1, itemtype = "Rasch") #Items from Dim2, not anchored


## D) Common Ruler ---------------------------------------------------

if(x["Equate"] == 1) {
  Data_F1 <- Data_F1
  Data_F2 <- Data_F2
}
if(x["Equate"] == 2) {
  Data_F1 <- rowSums(Data_F1)
  Data_F2 <- rowSums(Data_F2)
}

mod_common <- mirt(cbind(Data_F1, Data_F2), 1, itemtype = "Rasch")
Coef <- coef(mod_common, simplify = TRUE) #to get the anchoring values

# E) True Score Equating-------------------------------------------------------

#Form X - get the item parameter
mod1_anchor <- mirt(Data_F1, 1, itemtype = "Rasch", pars = "values")

if("d" %in% mod1_anchor[, "name"]) {
  mod1_anchor[
    which(
    mod1_anchor[,"name"] == "d"),
    "value"] <- Coef$items[
      which(
      is.na(Coef$items[1:x["nvar"], "d"]) == FALSE), "d"]
}

mod1_anchor[which(
  mod1_anchor[,"name"] == "d1"), "value"] <- Coef$items[which(
    is.na(Coef$items[1:x["nvar"], "d1"]) == FALSE), "d1"] #fix 1. threshold

mod1_anchor[which(
  mod1_anchor[,"name"] == "d2"), "value"] <- Coef$items[which(
```

```
      is.na(Coef$items[1:x["nvar"], "d2"]) == FALSE), "d2"] #fix 2. threshold

  mod1_anchor$est <- FALSE   #has to be set to false

 #anchored analysis
  mod1_F1 <- mirt(Data_F1, 1, itemtype = "Rasch", pars = mod1_anchor)


  # Form Y - get the item parameter
  mod2_anchor <- mirt(Data_F2, 1, itemtype = "Rasch", pars = "values")

  if("d" %in% mod2_anchor[, "name"]){
  mod2_anchor[which(
    mod2_anchor[,"name"] == "d"),
    "value"] <- Coef$items[((1:x["nvar"]) + x["nvar"])[which(
      is.na(Coef$items[(1:x["nvar"]) + x["nvar"], "d"]) == FALSE)],
      "d"]
  }

  mod2_anchor[which(mod2_anchor[,"name"] == "d1"), "value"] <-
    Coef$items[ ((1:x["nvar"]) + x["nvar"])[which(
      is.na(Coef$items[(1:x["nvar"]) + x["nvar"], "d1"]) == FALSE)], "d1"] #fix 1. threshold
  mod2_anchor[which(mod2_anchor[,"name"] == "d2"), "value"] <-
    Coef$items[ ((1:x["nvar"]) + x["nvar"])[which(
      is.na(Coef$items[(1:x["nvar"]) + x["nvar"], "d2"]) == FALSE)], "d2"] #fix 2. threshold
  mod2_anchor$est <- FALSE

  mod2_F2 <- mirt(Data_F2, 1, itemtype = "Rasch", pars = mod2_anchor) #anchored analysis



# F) Concordance Tables---------

  Theta_estimated_mod1 <- fscores(mod1_F1, method = TypAlgo,
                                  full.scores.SE = TRUE, verbose = FALSE)
  Theta_estimated_mod2 <- fscores(mod2_F2, method = TypAlgo,
                                  full.scores.SE = TRUE, verbose = FALSE)

  Theta1 <- Theta_estimated_mod1[, "F1"]
  Theta2 <- Theta_estimated_mod2[, "F1"]

  Theta1[which(Theta1 %in% c("-Inf", "Inf"))] <- NA
  Theta2[which(Theta2 %in% c("-Inf", "Inf"))] <- NA
```

```r
# Equating-------------------------

  Conversion1 <- cbind(Rsum = Rsum_F1, round(Theta_estimated_mod1[, 1], 3))
  Conversion2 <- cbind(Rsum = Rsum_F2, round(Theta_estimated_mod2[, 1], 3))

  Conv1 <- as.data.frame(unique(Conversion1[order(Conversion1[, 1]),]))
  Conv2 <- as.data.frame(unique(Conversion2[order(Conversion2[, 1]),]))

  Conv0 <- matrix(rep(0:(x["nvar"] * 2), 2),
                  ncol = 2, dimnames = list(c(1:(x["nvar"] * 2 + 1)),
                                           c("X", "Rsum")))

  Converse0 <- as.data.frame(merge(Conv0, Conv1, by = "Rsum", all = TRUE))
  Converse1 <- as.data.frame(merge(Converse0, Conv2, by = "Rsum", all = TRUE))

  Converse <- Converse1[, -which(colnames(Converse1) == "X")]

  Converse[which(Converse[, 2] %in% c("-Inf", "Inf")), 2] <- NA
  Converse[which(Converse[, 3] %in% c("-Inf", "Inf")), 3] <- NA


  Converse_Interpl <- Converse
  if(sum(is.na( Converse_Interpl[, 2])) > 0){
    Converse_Interpl[which(
      is.na(Converse_Interpl[, 2])), 2] <- interpolna(Converse_Interpl[, 2])
  }

  if(sum(is.na(Converse_Interpl[, 3])) > 0){
    Converse_Interpl[which(
      is.na(Converse_Interpl[, 3])), 3] <- interpolna(Converse_Interpl[, 3])
  }



  #B2) DGP - validation-------------------------


  #X to Y : now x is just the total score and we base the transformation on the
  #score values from the transformation table
  transform_X_to_Y <- function(X) {
  if(is.na(X) == FALSE) {
  Converse_Interpl[
      which.min(
        abs(
          Converse_Interpl[which(Converse_Interpl[,"Rsum"] %in% X), "V2.x"] -
            Converse_Interpl[, "V2.y"])), "Rsum"]
```

```r
  } else {
    return(NA)
  }
}


transform_Y_to_X <- function(X) {
  if(is.na(X) == FALSE) {
    Converse_Interpl[
      which.min(
        abs(
          Converse_Interpl[which(Converse_Interpl[, "Rsum"] %in% X), "V2.y"] -
            Converse_Interpl[, "V2.x"])), "Rsum"]
  } else {
    return(NA)
  }
}


Rsum_F2_Hat_original <- list()
Rsum_F1_Hat_original <- list()


  for(i in 1:x["n"]) {

  Rsum_F2_Hat_original[[i]] <- transform_X_to_Y(Rsum_F1[i])
  Rsum_F1_Hat_original[[i]] <- transform_Y_to_X(Rsum_F2[i])
  }


Conversions_X_to_Y <- cbind(Rsum_F2 = Rsum_F2,
                           Rsum_F2_Hat = do.call(
                             c, Rsum_F2_Hat_original))
Conversions_Y_to_X <- cbind(Rsum_F1 = Rsum_F1,
                            Rsum_F1_Hat = do.call(
                              c, Rsum_F1_Hat_original))


#H) WrappingUp ----------------------------------------------------------------


## Intuitive syntax for the formatting and
## saving the relevant results code lines are not included here.
## This part included, analysis of each output
## to extract the item fit, LID, targeting, equity, dimensionality
```

```r
  ## of the datasets at each step of the equating.
  ## If needed, the first author can be contacted.



 #..Thetas--------------------------------------------------

   ##Thetas, scores and equated scores to compute NRMSE and relative bias
   ##

 if(Save_Thetas == TRUE) {
  ##save the thetas, scores and transformed scores for analysis.
  All_the_Thetas <- cbind(Rsum_F1,
                       do.call(c, Rsum_F1_Hat_original),
                       Rsum_F2,
                       do.call(c, Rsum_F2_Hat_original),
                       Theta_mod1_start, Theta_mod2_start,
                       Theta_estimated_mod1, Theta_estimated_mod2)

  colnames(All_the_Thetas) <- c("Rsum_F1", "Rsum_F1_hat",
                             "Rsum_F2", "Rsum_F2_hat",
                             "F1_start", "SE_F1_start",
                             "F2_start", "SE_F2_start",
                             "F1_equated", "SE_F1_equated",
                             "F2_equated", "SE_F2_equated")
  }


  return(x)

}


# A.1. Parallelization -------------------------------------

#..NbrCluster ---------------

   cl <- makeCluster(..)
   registerDoParallel(cl)

foreach(k = 1:nrow(Combis_Grid)) %dopar% {


#..Libraries
      library(mirt)
      library(yarrr)
```

```
  library(splines)
  library(purrr)
  library(effectsize)
  library(grDevices)
  library(pBrackets)

  Equate_Sim(Combis_Grid[k,],
             Save_Thetas = TRUE, algoType = TypAlgo)

  }

stopCluster(cl)
```

Appendix C

Table 1: Mean(SD) the factor correlation, mean(SD) difficulty shift and mean(SD) difficulty dispersion in simulation settings with a person parameter dispersion of $\sigma_\theta = 0.5$, for the two-dimensional PCM analyses aggregated for the 500 replications.

**Simulation Input**			**Simulation Result**			
Factor	IP Distribution		Factor Correlation			
Correlation	$(\mu_\delta; \sigma_\delta)$		I=10 &	I=20 &	I=10 &	I=20 &
ρ_{sim}	Form X	Form Y	N=500	N=500	N=1000	N=1000
0.25	(0,1)	(0,1)	0.56 (0.09)	0.71 (0.04)	0.57 (0.06)	0.71 (0.03)
	(0,1)	(0,2)	0.51 (0.11)	0.7 (0.04)	0.52 (0.08)	0.7 (0.03)
	(0,2)	(0,2)	0.45 (0.14)	0.68 (0.05)	0.46 (0.09)	0.68 (0.04)
	(-0.5,1)	(0.5,1)	0.55 (0.09)	0.71 (0.06)	0.55 (0.06)	0.71 (0.03)
	(-0.5,1)	(0.5,2)	0.49 (0.12)	0.69 (0.07)	0.5 (0.08)	0.7 (0.03)
	(-0.5,2)	(0.5,2)	0.44 (0.14)	0.67 (0.07)	0.45 (0.1)	0.68 (0.04)
	(-1,1)	(1,1)	0.51 (0.12)	0.61 (0.3)	0.51 (0.08)	0.66 (0.2)
	(-1,1)	(1,2)	0.46 (0.13)	0.66 (0.16)	0.46 (0.09)	0.68 (0.06)
	(-1,2)	(1,2)	0.42 (0.13)	0.66 (0.08)	0.42 (0.1)	0.66 (0.08)
0.5	(0,1)	(0,1)	0.72 (0.05)	0.82 (0.02)	0.72 (0.04)	0.82 (0.02)
	(0,1)	(0,2)	0.68 (0.06)	0.81 (0.03)	0.69 (0.05)	0.81 (0.02)
	(0,2)	(0,2)	0.65 (0.07)	0.79 (0.03)	0.65 (0.06)	0.79 (0.02)
	(-0.5,1)	(0.5,1)	0.71 (0.05)	0.81 (0.02)	0.71 (0.04)	0.81 (0.02)
	(-0.5,1)	(0.5,2)	0.67 (0.06)	0.81 (0.03)	0.68 (0.05)	0.8 (0.02)
	(-0.5,2)	(0.5,2)	0.64 (0.08)	0.79 (0.03)	0.64 (0.06)	0.79 (0.02)
	(-1,1)	(1,1)	0.68 (0.06)	0.8 (0.03)	0.68 (0.05)	0.8 (0.02)
	(-1,1)	(1,2)	0.65 (0.07)	0.8 (0.03)	0.65 (0.06)	0.8 (0.02)
	(-1,2)	(1,2)	0.62 (0.08)	0.78 (0.04)	0.62 (0.07)	0.78 (0.02)
0.75	(0,1)	(0,1)	0.83 (0.03)	0.9 (0.02)	0.82 (0.02)	0.9 (0.01)
	(0,1)	(0,2)	0.8 (0.04)	0.89 (0.02)	0.8 (0.03)	0.89 (0.01)
	(0,2)	(0,2)	0.77 (0.05)	0.88 (0.02)	0.77 (0.04)	0.88 (0.02)
	(-0.5,1)	(0.5,1)	0.82 (0.03)	0.9 (0.02)	0.82 (0.02)	0.9 (0.01)
	(-0.5,1)	(0.5,2)	0.79 (0.04)	0.89 (0.02)	0.79 (0.03)	0.89 (0.01)
	(-0.5,2)	(0.5,2)	0.77 (0.05)	0.88 (0.02)	0.76 (0.04)	0.88 (0.02)
	(-1,1)	(1,1)	0.8 (0.04)	0.89 (0.02)	0.8 (0.03)	0.89 (0.01)
	(-1,1)	(1,2)	0.78 (0.05)	0.88 (0.02)	0.78 (0.04)	0.89 (0.01)
	(-1,2)	(1,2)	0.75 (0.06)	0.87 (0.02)	0.75 (0.05)	0.88 (0.02)
1	(0,1)	(0,1)	0.9 (0.02)	0.96 (0.01)	0.9 (0.01)	0.96 (0)
	(0,1)	(0,2)	0.88 (0.02)	0.95 (0.01)	0.88 (0.02)	0.96 (0.01)
	(0,2)	(0,2)	0.86 (0.03)	0.94 (0.01)	0.86 (0.03)	0.95 (0.01)
	(-0.5,1)	(0.5,1)	0.89 (0.02)	0.95 (0.01)	0.9 (0.01)	0.96 (0)
	(-0.5,1)	(0.5,2)	0.87 (0.03)	0.95 (0.01)	0.88 (0.02)	0.95 (0.01)
	(-0.5,2)	(0.5,2)	0.85 (0.03)	0.94 (0.01)	0.85 (0.03)	0.94 (0.01)
	(-1,1)	(1,1)	0.88 (0.02)	0.95 (0.01)	0.88 (0.02)	0.95 (0.01)
	(-1,1)	(1,2)	0.86 (0.03)	0.95 (0.01)	0.86 (0.03)	0.95 (0.01)
	(-1,2)	(1,2)	0.84 (0.04)	0.94 (0.01)	0.84 (0.04)	0.94 (0.01)

Table 2: Mean(SD) the factor correlation, mean(SD) difficulty shift and mean(SD) difficulty dispersion in simulation settings with a person parameter dispersion of $\sigma_\theta = 1$, for the two-dimensional PCM analyses aggregated for the 500 replications.

**Simulation Input**			**Simulation Result**			
Factor	IP Distribution		Factor Correlation			
Correlation	$(\mu_\delta; \sigma_\delta)$		I=10 &	I=20 &	I=10 &	I=20 &
ρ_{sim}	Form X	Form Y	N=500	N=500	N=1000	N=1000
	(0,1)	(0,1)	0.25 (0.05)	0.25 (0.05)	0.25 (0.04)	0.25 (0.03)
0.25	(0,1)	(0,2)	0.25 (0.06)	0.25 (0.05)	0.25 (0.04)	0.25 (0.03)
	(0,2)	(0,2)	0.25 (0.06)	0.25 (0.05)	0.25 (0.04)	0.25 (0.03)
	(-0.5,1)	(0.5,1)	0.25 (0.05)	0.25 (0.05)	0.25 (0.04)	0.25 (0.03)
	(-0.5,1)	(0.5,2)	0.25 (0.06)	0.25 (0.05)	0.25 (0.04)	0.25 (0.03)
	(-0.5,2)	(0.5,2)	0.25 (0.06)	0.25 (0.05)	0.25 (0.04)	0.25 (0.03)
	(-1,1)	(1,1)	0.25 (0.06)	0.25 (0.05)	0.25 (0.04)	0.25 (0.03)
	(-1,1)	(1,2)	0.25 (0.06)	0.25 (0.05)	0.25 (0.04)	0.25 (0.04)
	(-1,2)	(1,2)	0.25 (0.06)	0.25 (0.05)	0.25 (0.04)	0.25 (0.03)
	(0,1)	(0,1)	0.5 (0.04)	0.5 (0.04)	0.5 (0.03)	0.5 (0.03)
0.5	(0,1)	(0,2)	0.5 (0.05)	0.5 (0.04)	0.5 (0.03)	0.5 (0.03)
	(0,2)	(0,2)	0.5 (0.05)	0.5 (0.04)	0.5 (0.04)	0.5 (0.03)
	(-0.5,1)	(0.5,1)	0.5 (0.05)	0.5 (0.04)	0.5 (0.03)	0.5 (0.03)
	(-0.5,1)	(0.5,2)	0.5 (0.05)	0.5 (0.04)	0.5 (0.03)	0.5 (0.03)
	(-0.5,2)	(0.5,2)	0.5 (0.05)	0.5 (0.04)	0.5 (0.03)	0.5 (0.03)
	(-1,1)	(1,1)	0.5 (0.05)	0.5 (0.04)	0.5 (0.03)	0.5 (0.03)
	(-1,1)	(1,2)	0.5 (0.05)	0.5 (0.04)	0.5 (0.03)	0.5 (0.03)
	(-1,2)	(1,2)	0.5 (0.05)	0.5 (0.04)	0.49 (0.03)	0.5 (0.03)
	(0,1)	(0,1)	0.74 (0.03)	0.75 (0.02)	0.74 (0.02)	0.75 (0.02)
0.75	(0,1)	(0,2)	0.74 (0.03)	0.75 (0.03)	0.74 (0.02)	0.75 (0.02)
	(0,2)	(0,2)	0.74 (0.04)	0.75 (0.03)	0.74 (0.03)	0.75 (0.02)
	(-0.5,1)	(0.5,1)	0.74 (0.03)	0.75 (0.02)	0.74 (0.02)	0.75 (0.02)
	(-0.5,1)	(0.5,2)	0.74 (0.04)	0.75 (0.03)	0.74 (0.02)	0.75 (0.02)
	(-0.5,2)	(0.5,2)	0.74 (0.04)	0.75 (0.03)	0.74 (0.02)	0.75 (0.02)
	(-1,1)	(1,1)	0.74 (0.04)	0.75 (0.03)	0.74 (0.02)	0.75 (0.02)
	(-1,1)	(1,2)	0.73 (0.04)	0.75 (0.03)	0.74 (0.02)	0.75 (0.02)
	(-1,2)	(1,2)	0.73 (0.04)	0.75 (0.03)	0.73 (0.03)	0.75 (0.02)
	(0,1)	(0,1)	0.93 (0.01)	0.96 (0.01)	0.93 (0.01)	0.96 (0)
1	(0,1)	(0,2)	0.93 (0.01)	0.96 (0.01)	0.92 (0.01)	0.96 (0)
	(0,2)	(0,2)	0.92 (0.02)	0.96 (0.01)	0.92 (0.01)	0.96 (0.01)
	(-0.5,1)	(0.5,1)	0.93 (0.01)	0.96 (0.01)	0.93 (0.01)	0.96 (0)
	(-0.5,1)	(0.5,2)	0.93 (0.01)	0.96 (0.01)	0.92 (0.01)	0.96 (0)
	(-0.5,2)	(0.5,2)	0.92 (0.02)	0.96 (0.01)	0.92 (0.01)	0.96 (0)
	(-1,1)	(1,1)	0.93 (0.01)	0.96 (0.01)	0.92 (0.01)	0.96 (0)
	(-1,1)	(1,2)	0.92 (0.02)	0.96 (0.01)	0.92 (0.01)	0.96 (0)
	(-1,2)	(1,2)	0.91 (0.02)	0.95 (0.01)	0.91 (0.01)	0.96 (0.01)

Table 3: Mean(SD) the factor correlation, mean(SD) difficulty shift and mean(SD) difficulty dispersion in simulation settings with a person parameter dispersion of $\sigma_\theta = 2$, for the two-dimensional PCM analyses aggregated for the 500 replications.

**Simulation Input**			**Simulation Result**			
Factor	IP Distribution		Factor Correlation			
Correlation	($\mu_\delta; \sigma_\delta$)		I=10 &	I=20 &	I=10 &	I=20 &
ρ_{sim}	Form X	Form Y	N=500	N=500	N=1000	N=1000
	(0,1)	(0,1)	0.2 (0.04)	0.18 (0.03)	0.2 (0.03)	0.17 (0.02)
0.25	(0,1)	(0,2)	0.2 (0.04)	0.18 (0.03)	0.2 (0.03)	0.18 (0.02)
	(0,2)	(0,2)	0.2 (0.04)	0.18 (0.03)	0.2 (0.03)	0.18 (0.02)
	(-0.5,1)	(0.5,1)	0.2 (0.04)	0.18 (0.03)	0.2 (0.03)	0.18 (0.02)
	(-0.5,1)	(0.5,2)	0.2 (0.04)	0.18 (0.03)	0.2 (0.03)	0.18 (0.02)
	(-0.5,2)	(0.5,2)	0.2 (0.04)	0.18 (0.03)	0.2 (0.03)	0.18 (0.02)
	(-1,1)	(1,1)	0.2 (0.04)	0.18 (0.03)	0.2 (0.03)	0.18 (0.02)
	(-1,1)	(1,2)	0.2 (0.04)	0.18 (0.03)	0.2 (0.03)	0.18 (0.02)
	(-1,2)	(1,2)	0.21 (0.04)	0.18 (0.03)	0.21 (0.03)	0.18 (0.02)
	(0,1)	(0,1)	0.41 (0.04)	0.37 (0.03)	0.41 (0.03)	0.37 (0.02)
0.5	(0,1)	(0,2)	0.42 (0.04)	0.37 (0.03)	0.42 (0.03)	0.37 (0.02)
	(0,2)	(0,2)	0.43 (0.04)	0.38 (0.03)	0.43 (0.03)	0.38 (0.02)
	(-0.5,1)	(0.5,1)	0.41 (0.04)	0.37 (0.03)	0.41 (0.03)	0.37 (0.02)
	(-0.5,1)	(0.5,2)	0.42 (0.04)	0.38 (0.03)	0.42 (0.03)	0.37 (0.02)
	(-0.5,2)	(0.5,2)	0.43 (0.04)	0.38 (0.03)	0.43 (0.03)	0.38 (0.02)
	(-1,1)	(1,1)	0.42 (0.04)	0.37 (0.03)	0.42 (0.03)	0.37 (0.02)
	(-1,1)	(1,2)	0.42 (0.04)	0.38 (0.03)	0.42 (0.03)	0.38 (0.02)
	(-1,2)	(1,2)	0.43 (0.04)	0.38 (0.03)	0.43 (0.03)	0.38 (0.02)
	(0,1)	(0,1)	0.67 (0.03)	0.62 (0.03)	0.67 (0.02)	0.61 (0.02)
0.75	(0,1)	(0,2)	0.68 (0.03)	0.62 (0.03)	0.68 (0.02)	0.62 (0.02)
	(0,2)	(0,2)	0.69 (0.03)	0.63 (0.03)	0.69 (0.02)	0.63 (0.02)
	(-0.5,1)	(0.5,1)	0.67 (0.03)	0.62 (0.03)	0.67 (0.02)	0.61 (0.02)
	(-0.5,1)	(0.5,2)	0.68 (0.03)	0.62 (0.03)	0.68 (0.02)	0.62 (0.02)
	(-0.5,2)	(0.5,2)	0.69 (0.03)	0.63 (0.03)	0.69 (0.02)	0.63 (0.02)
	(-1,1)	(1,1)	0.67 (0.03)	0.62 (0.03)	0.68 (0.02)	0.62 (0.02)
	(-1,1)	(1,2)	0.69 (0.03)	0.62 (0.03)	0.69 (0.02)	0.62 (0.02)
	(-1,2)	(1,2)	0.7 (0.03)	0.63 (0.03)	0.7 (0.02)	0.63 (0.02)
	(0,1)	(0,1)	0.95 (0)	0.96 (0.01)	0.96 (0.01)	0.96 (0)
1	(0,1)	(0,2)	0.95 (0.01)	0.96 (0.01)	0.96 (0.01)	0.96 (0)
	(0,2)	(0,2)	0.95 (0.01)	0.96 (0.01)	0.96 (0.01)	0.96 (0)
	(-0.5,1)	(0.5,1)	0.95 (0)	0.96 (0.01)	0.96 (0.01)	0.96 (0)
	(-0.5,1)	(0.5,2)	0.95 (0)	0.96 (0.01)	0.96 (0.01)	0.96 (0)
	(-0.5,2)	(0.5,2)	0.95 (0.01)	0.96 (0.01)	0.96 (0.01)	0.96 (0)
	(-1,1)	(1,1)	0.95 (0)	0.96 (0.01)	0.96 (0.01)	0.97 (0)
	(-1,1)	(1,2)	0.95 (0)	0.96 (0.01)	0.96 (0.01)	0.96 (0)
	(-1,2)	(1,2)	0.95 (0)	0.96 (0.01)	0.96 (0.01)	0.96 (0)

Table 4: Relative Bias when $I = 10$ and $N = 500$

**Simulation Input**				**Precision: Relative Bias Quantiles [Q1;Q3]**					
Cor.	Shift	IP (μ_δ, σ_δ)		PP $\sigma_\theta = 0.5$		PP $\sigma_\theta = 1$		PP $\sigma_\theta = 2$	
ρ_{sim}	Δ_{XY}	Scale X	Scale Y	X to Y	Y to X	X to Y	Y to X	X to Y	Y to X
0.25	0	(0,1)	(0,1)	[-0.9;0.83]	[-0.91;1.09]	[-0.83;0.68]	[-0.68;0.87]	[-1.44;1.22]	[-1.38;1.38]
		(0,1)	(0,2)	[-0.45;0.48]	[-0.55;0.59]	[-0.59;0.51]	[-0.64;0.66]	[-1.56;1.12]	[-1.16;1.41]
		(0,2)	(0,2)	[-0.77;0.74]	[-0.76;0.81]	[-0.85;0.63]	[-0.68;0.77]	[-1.49;1.05]	[-1.38;1.28]
	1	(-0.5,1)	(0.5,1)	[-1.61;0.66]	[-0.43;1.12]	[-2.75;-0.73]	[0.55;1.76]	[-5.18;-2.43]	[1.99;3.58]
		(-0.5,1)	(0.5,2)	[-0.73;0.57]	[-0.23;1.01]	[-2.01;-0.22]	[0.3;1.61]	[-4.69;-1.3]	[1.04;3.16]
		(-0.5,2)	(0.5,2)	[-1.02;0.81]	[-0.6;0.67]	[-1.86;0.17]	[-0.15;1.23]	[-3.49;-0.61]	[0.49;2.52]
	2	(-1,1)	(1,1)	[-2.5;-0.06]	[-0.07;1.12]	[-4.9;-2.39]	[1.21;2.25]	[-9.33;-6.39]	[3.74;5]
		(-1,1)	(1,2)	[-1.33;0.44]	[-0.03;1.2]	[-3.49;-1.08]	[0.85;1.97]	[-7.32;-3.85]	[2.46;3.96]
		(-1,2)	(1,2)	[-1.58;0.78]	[-0.38;0.78]	[-2.96;-0.49]	[0.24;1.48]	[-6.12;-2.6]	[1.57;3.2]
0.5	0	(0,1)	(0,1)	[-0.92;0.94]	[-0.84;1.05]	[-0.8;0.62]	[-0.62;0.75]	[-1;0.73]	[-0.76;0.9]
		(0,1)	(0,2)	[-0.49;0.52]	[-0.55;0.58]	[-0.51;0.42]	[-0.6;0.58]	[-1;0.7]	[-0.83;0.86]
		(0,2)	(0,2)	[-0.73;0.74]	[-0.8;0.83]	[-0.71;0.56]	[-0.62;0.63]	[-0.98;0.77]	[-0.93;0.84]
	1	(-0.5,1)	(0.5,1)	[-1.46;0.98]	[-0.55;0.95]	[-1.86;-0.06]	[0.08;1.26]	[-3.12;-1.21]	[1.02;2.18]
		(-0.5,1)	(0.5,2)	[-0.65;0.6]	[-0.3;0.84]	[-1.33;0.14]	[-0.02;1.14]	[-2.5;-0.55]	[0.5;1.87]
		(-0.5,2)	(0.5,2)	[-0.99;0.96]	[-0.69;0.64]	[-1.2;0.46]	[-0.29;0.88]	[-2.26;-0.15]	[0.07;1.55]
	2	(-1,1)	(1,1)	[-2.02;0.61]	[-0.3;0.95]	[-3.09;-0.96]	[0.41;1.46]	[-5.09;-3.01]	[1.77;2.94]
		(-1,1)	(1,2)	[-1.06;0.62]	[-0.15;1.02]	[-2.03;-0.26]	[0.36;1.43]	[-3.98;-1.58]	[1.25;2.37]
		(-1,2)	(1,2)	[-1.36;0.91]	[-0.46;0.77]	[-2.01;0.06]	[0;1.03]	[-3.54;-1.18]	[0.7;1.9]
0.75	0	(0,1)	(0,1)	[-0.89;0.96]	[-0.87;0.96]	[-0.76;0.73]	[-0.64;0.77]	[-0.56;0.47]	[-0.55;0.59]
		(0,1)	(0,2)	[-0.5;0.5]	[-0.59;0.64]	[-0.47;0.43]	[-0.59;0.56]	[-0.45;0.45]	[-0.49;0.52]
		(0,2)	(0,2)	[-0.75;0.88]	[-0.66;0.82]	[-0.67;0.61]	[-0.62;0.7]	[-0.65;0.48]	[-0.55;0.57]
	1	(-0.5,1)	(0.5,1)	[-1.18;1.16]	[-0.71;0.81]	[-0.96;0.58]	[-0.43;0.7]	[-1.05;0.17]	[-0.16;0.81]
		(-0.5,1)	(0.5,2)	[-0.48;0.73]	[-0.42;0.73]	[-0.72;0.45]	[-0.29;0.72]	[-0.77;0.42]	[-0.14;0.85]
		(-0.5,2)	(0.5,2)	[-0.94;1]	[-0.69;0.66]	[-0.85;0.69]	[-0.54;0.48]	[-0.92;0.39]	[-0.35;0.7]
	2	(-1,1)	(1,1)	[-1.45;1.02]	[-0.47;0.72]	[-1.37;0.55]	[-0.31;0.7]	[-1.21;0.51]	[-0.34;0.64]
		(-1,1)	(1,2)	[-0.8;1.05]	[-0.35;0.87]	[-0.93;0.57]	[-0.19;0.85]	[-0.76;0.93]	[-0.06;0.77]
		(-1,2)	(1,2)	[-1.19;1.14]	[-0.57;0.54]	[-1.22;0.76]	[-0.36;0.64]	[-1.19;0.48]	[-0.29;0.69]
1	0	(0,1)	(0,1)	[-0.9;1.07]	[-0.86;0.94]	[-0.79;0.77]	[-0.79;0.82]	[-0.63;0.68]	[-0.65;0.67]
		(0,1)	(0,2)	[-0.44;0.47]	[-0.75;0.52]	[-0.48;0.5]	[-0.62;0.6]	[-0.55;0.62]	[-0.65;0.51]
		(0,2)	(0,2)	[-0.76;0.75]	[-0.85;0.66]	[-0.69;0.59]	[-0.6;0.76]	[-0.6;0.73]	[-0.65;0.6]
	1	(-0.5,1)	(0.5,1)	[-1.07;1.31]	[-0.82;0.78]	[-0.22;1.32]	[-0.91;0.22]	[0.67;2.08]	[-1.59;-0.62]
		(-0.5,1)	(0.5,2)	[-0.4;0.94]	[-0.45;0.68]	[-0.26;0.93]	[-0.71;0.32]	[0.55;2.01]	[-1.15;-0.03]
		(-0.5,2)	(0.5,2)	[-0.77;0.95]	[-0.71;0.65]	[-0.59;0.97]	[-0.72;0.53]	[-0.11;1.35]	[-0.98;0.05]
	2	(-1,1)	(1,1)	[-1.09;1.67]	[-0.71;0.45]	[0.28;2.1]	[-1.04;-0.12]	[2.65;5.01]	[-2.8;-1.62]
		(-1,1)	(1,2)	[-0.63;1.31]	[-0.58;0.65]	[0.05;1.74]	[-0.8;0.25]	[1.78;4.54]	[-1.82;-0.59]
		(-1,2)	(1,2)	[-1.07;1.41]	[-0.74;0.53]	[-0.62;1.49]	[-0.79;0.27]	[0.54;2.58]	[-1.53;-0.36]

IP: Item Parameter; PP: Person Parameter

Table 5: Relative Bias when $I = 20$ and $N = 500$

**Simulation Input**				**Precision: Relative Bias Quantiles [Q1;Q3]**					
Cor.	Shift	IP (μ_δ, σ_δ)		PP $\sigma_\theta = 0.5$		PP $\sigma_\theta = 1$		PP $\sigma_\theta = 2$	
ρ_{sim}	Δ_{XY}	Scale X	Scale Y	X to Y	Y to X	X to Y	Y to X	X to Y	Y to X
0.25	0	(0,1)	(0,1)	[-0.36;0.36]	[-0.32;0.35]	[-0.55;0.45]	[-0.48;0.49]	[-1.07;1.04]	[-1.05;1.02]
		(0,1)	(0,2)	[-0.17;0.15]	[-0.23;0.23]	[-0.37;0.35]	[-0.46;0.41]	[-0.97;0.88]	[-0.91;0.89]
		(0,2)	(0,2)	[-0.29;0.27]	[-0.25;0.38]	[-0.43;0.37]	[-0.42;0.37]	[-1.07;1.05]	[-1.16;1.04]
	1	(-0.5,1)	(0.5,1)	[-1.07;-0.11]	[0.11;0.72]	[-2.53;-1.31]	[1;1.69]	[-5.58;-3.22]	[2.65;3.92]
		(-0.5,1)	(0.5,2)	[-0.44;0.03]	[0.14;0.57]	[-1.71;-0.61]	[0.64;1.38]	[-4.58;-2.02]	[1.63;3.07]
		(-0.5,2)	(0.5,2)	[-0.6;0.2]	[-0.14;0.44]	[-1.38;-0.18]	[0.15;1]	[-3.54;-1.15]	[0.98;2.51]
	2	(-1,1)	(1,1)	[-1.94;-0.72]	[0.37;0.88]	[-5.3;-3.61]	[1.79;2.44]	[-11.15;-8.11]	[4.97;6.06]
		(-1,1)	(1,2)	[-1.02;-0.21]	[0.32;0.77]	[-3.39;-1.93]	[1.28;1.9]	[-8.65;-5.5]	[3.26;4.59]
		(-1,2)	(1,2)	[-0.92;0.09]	[-0.05;0.51]	[-2.5;-0.96]	[0.62;1.36]	[-6.53;-3.47]	[2.39;3.66]
0.5	0	(0,1)	(0,1)	[-0.35;0.37]	[-0.31;0.36]	[-0.43;0.36]	[-0.36;0.4]	[-0.66;0.65]	[-0.75;0.63]
		(0,1)	(0,2)	[-0.17;0.13]	[-0.18;0.2]	[-0.26;0.23]	[-0.33;0.32]	[-0.67;0.61]	[-0.65;0.68]
		(0,2)	(0,2)	[-0.34;0.27]	[-0.23;0.33]	[-0.27;0.28]	[-0.3;0.29]	[-0.77;0.7]	[-0.72;0.69]
	1	(-0.5,1)	(0.5,1)	[-0.86;0.13]	[-0.07;0.57]	[-1.72;-0.72]	[0.59;1.15]	[-3.64;-2.17]	[1.7;2.6]
		(-0.5,1)	(0.5,2)	[-0.33;0.09]	[0.03;0.45]	[-1.15;-0.33]	[0.37;0.92]	[-2.97;-1.25]	[1.01;2.03]
		(-0.5,2)	(0.5,2)	[-0.38;0.27]	[-0.19;0.32]	[-0.95;-0.03]	[0.09;0.7]	[-2.38;-0.73]	[0.63;1.69]
	2	(-1,1)	(1,1)	[-1.38;-0.29]	[0.16;0.65]	[-3.45;-2.12]	[1.08;1.64]	[-7.19;-5.16]	[3.14;3.92]
		(-1,1)	(1,2)	[-0.74;0.05]	[0.15;0.66]	[-2.16;-1.1]	[0.79;1.28]	[-5.48;-3.36]	[2.09;2.92]
		(-1,2)	(1,2)	[-0.71;0.25]	[-0.12;0.41]	[-1.75;-0.59]	[0.32;0.92]	[-4.12;-2.28]	[1.55;2.38]
0.75	0	(0,1)	(0,1)	[-0.36;0.32]	[-0.32;0.38]	[-0.32;0.27]	[-0.28;0.35]	[-0.34;0.34]	[-0.37;0.32]
		(0,1)	(0,2)	[-0.13;0.16]	[-0.19;0.22]	[-0.16;0.17]	[-0.17;0.21]	[-0.33;0.3]	[-0.37;0.33]
		(0,2)	(0,2)	[-0.31;0.27]	[-0.24;0.28]	[-0.23;0.22]	[-0.24;0.26]	[-0.37;0.35]	[-0.36;0.39]
	1	(-0.5,1)	(0.5,1)	[-0.64;0.35]	[-0.2;0.44]	[-0.86;-0.15]	[0.14;0.6]	[-1.62;-0.84]	[0.64;1.12]
		(-0.5,1)	(0.5,2)	[-0.2;0.23]	[-0.04;0.34]	[-0.53;-0.04]	[0.08;0.5]	[-1.27;-0.46]	[0.39;0.95]
		(-0.5,2)	(0.5,2)	[-0.35;0.35]	[-0.25;0.28]	[-0.53;0.14]	[-0.09;0.42]	[-1.04;-0.23]	[0.24;0.78]
	2	(-1,1)	(1,1)	[-0.9;0.25]	[-0.03;0.42]	[-1.58;-0.57]	[0.32;0.78]	[-2.9;-1.79]	[1.06;1.62]
		(-1,1)	(1,2)	[-0.46;0.28]	[0.02;0.49]	[-1.06;-0.24]	[0.29;0.72]	[-2.08;-1.06]	[0.83;1.29]
		(-1,2)	(1,2)	[-0.5;0.42]	[-0.21;0.28]	[-0.89;-0.03]	[0.04;0.47]	[-1.73;-0.85]	[0.56;1.04]
1	0	(0,1)	(0,1)	[-0.4;0.41]	[-0.4;0.44]	[-0.39;0.37]	[-0.35;0.44]	[-0.26;0.31]	[-0.28;0.29]
		(0,1)	(0,2)	[-0.15;0.16]	[-0.21;0.18]	[-0.16;0.15]	[-0.14;0.21]	[-0.23;0.23]	[-0.2;0.22]
		(0,2)	(0,2)	[-0.29;0.27]	[-0.23;0.33]	[-0.27;0.26]	[-0.23;0.27]	[-0.22;0.25]	[-0.22;0.22]
	1	(-0.5,1)	(0.5,1)	[-0.39;0.56]	[-0.36;0.27]	[-0.05;0.55]	[-0.36;0.04]	[0.44;0.96]	[-0.74;-0.32]
		(-0.5,1)	(0.5,2)	[-0.07;0.36]	[-0.18;0.22]	[-0.06;0.39]	[-0.26;0.1]	[0.29;0.86]	[-0.48;-0.1]
		(-0.5,2)	(0.5,2)	[-0.28;0.42]	[-0.29;0.24]	[-0.21;0.38]	[-0.28;0.19]	[0.03;0.55]	[-0.39;-0.01]
	2	(-1,1)	(1,1)	[-0.35;0.69]	[-0.26;0.17]	[0.15;0.97]	[-0.42;-0.03]	[1.34;2.3]	[-1.26;-0.75]
		(-1,1)	(1,2)	[-0.2;0.55]	[-0.16;0.32]	[0;0.63]	[-0.28;0.11]	[0.92;1.91]	[-0.72;-0.32]
		(-1,2)	(1,2)	[-0.33;0.61]	[-0.32;0.2]	[-0.08;0.6]	[-0.3;0.1]	[0.29;0.98]	[-0.6;-0.18]

IP: Item Parameter; PP: Person Parameter

Table 6: Relative Bias when $I = 10$ and $N = 1000$

**Simulation Input**				**Precision: Relative Bias Quantiles [Q1;Q3]**					
Cor.	Shift	IP (μ_δ, σ_δ)		PP $\sigma_\theta = 0.5$		PP $\sigma_\theta = 1$		PP $\sigma_\theta = 2$	
ρ_{sim}	Δ_{XY}	Scale X	Scale Y	X to Y	Y to X	X to Y	Y to X	X to Y	Y to X
0.25	0	(0,1)	(0,1)	[-0.89;0.93]	[-0.96;0.98]	[-0.87;0.76]	[-0.71;0.77]	[-1.52;1.23]	[-1.35;1.45]
		(0,1)	(0,2)	[-0.46;0.46]	[-0.64;0.5]	[-0.64;0.42]	[-0.63;0.63]	[-1.49;1.1]	[-1.17;1.39]
		(0,2)	(0,2)	[-0.85;0.7]	[-0.55;0.72]	[-0.74;0.65]	[-0.72;0.67]	[-1.64;1.17]	[-1.42;1.42]
	1	(-0.5,1)	(0.5,1)	[-1.64;0.71]	[-0.42;0.98]	[-2.68;-0.77]	[0.57;1.77]	[-5.12;-2.44]	[2.05;3.49]
		(-0.5,1)	(0.5,2)	[-0.7;0.55]	[-0.26;0.92]	[-1.87;-0.16]	[0.29;1.49]	[-4.49;-1.34]	[1.28;3.07]
		(-0.5,2)	(0.5,2)	[-1.22;0.69]	[-0.47;0.8]	[-1.86;0.17]	[-0.12;1.26]	[-3.6;-0.71]	[0.55;2.52]
	2	(-1,1)	(1,1)	[-2.82;0.06]	[0.05;1.23]	[-4.67;-2.52]	[1.26;2.23]	[-9.57;-6.39]	[3.84;4.99]
		(-1,1)	(1,2)	[-1.3;0.55]	[0.01;1.22]	[-3.36;-1.05]	[0.82;2.05]	[-7.6;-3.65]	[2.66;4.05]
		(-1,2)	(1,2)	[-1.48;0.79]	[-0.41;0.78]	[-2.94;-0.49]	[0.22;1.46]	[-6.18;-2.52]	[1.67;3.38]
0.5	0	(0,1)	(0,1)	[-0.93;0.98]	[-0.94;0.92]	[-0.71;0.68]	[-0.64;0.72]	[-1.04;0.82]	[-0.9;0.91]
		(0,1)	(0,2)	[-0.43;0.55]	[-0.68;0.6]	[-0.47;0.41]	[-0.54;0.51]	[-0.99;0.71]	[-0.85;0.87]
		(0,2)	(0,2)	[-0.81;0.71]	[-0.6;0.79]	[-0.61;0.54]	[-0.65;0.63]	[-1.03;0.8]	[-0.88;0.97]
	1	(-0.5,1)	(0.5,1)	[-1.33;0.89]	[-0.56;0.92]	[-1.91;-0.17]	[0.08;1.16]	[-3.06;-1.34]	[1.04;2.25]
		(-0.5,1)	(0.5,2)	[-0.58;0.69]	[-0.34;0.8]	[-1.1;0.16]	[0.01;0.99]	[-2.75;-0.6]	[0.64;1.87]
		(-0.5,2)	(0.5,2)	[-1.21;0.76]	[-0.53;0.76]	[-1.44;0.37]	[-0.27;0.94]	[-2.32;-0.23]	[0.2;1.53]
	2	(-1,1)	(1,1)	[-2.23;0.6]	[-0.22;0.96]	[-3.16;-1.04]	[0.51;1.41]	[-5.34;-3.21]	[1.93;2.87]
		(-1,1)	(1,2)	[-1.03;0.73]	[-0.18;1.05]	[-2.11;-0.25]	[0.4;1.51]	[-4.13;-1.69]	[1.32;2.36]
		(-1,2)	(1,2)	[-1.29;1.04]	[-0.58;0.73]	[-2.1;0.07]	[-0.06;1.01]	[-3.46;-1.16]	[0.76;1.94]
0.75	0	(0,1)	(0,1)	[-0.96;1.08]	[-1.04;0.86]	[-0.7;0.76]	[-0.63;0.72]	[-0.58;0.53]	[-0.58;0.53]
		(0,1)	(0,2)	[-0.45;0.5]	[-0.66;0.52]	[-0.43;0.47]	[-0.53;0.48]	[-0.54;0.44]	[-0.45;0.47]
		(0,2)	(0,2)	[-0.77;0.66]	[-0.6;0.72]	[-0.62;0.56]	[-0.56;0.62]	[-0.49;0.52]	[-0.64;0.54]
	1	(-0.5,1)	(0.5,1)	[-1.23;1.09]	[-0.65;0.73]	[-1.02;0.62]	[-0.4;0.64]	[-0.92;0.23]	[-0.11;0.73]
		(-0.5,1)	(0.5,2)	[-0.43;0.77]	[-0.44;0.72]	[-0.6;0.51]	[-0.33;0.66]	[-0.8;0.43]	[-0.12;0.77]
		(-0.5,2)	(0.5,2)	[-1.01;0.88]	[-0.63;0.69]	[-0.95;0.68]	[-0.41;0.69]	[-1;0.42]	[-0.31;0.66]
	2	(-1,1)	(1,1)	[-1.68;1.1]	[-0.42;0.75]	[-1.39;0.53]	[-0.24;0.66]	[-1.13;0.46]	[-0.27;0.55]
		(-1,1)	(1,2)	[-0.8;1.08]	[-0.32;0.85]	[-0.97;0.6]	[-0.11;0.82]	[-0.75;0.83]	[-0.1;0.85]
		(-1,2)	(1,2)	[-1.03;1.22]	[-0.6;0.57]	[-1.16;0.6]	[-0.42;0.65]	[-1.18;0.32]	[-0.26;0.66]
1	0	(0,1)	(0,1)	[-1.04;0.85]	[-0.86;1.08]	[-0.82;0.77]	[-0.76;0.83]	[-0.65;0.74]	[-0.6;0.67]
		(0,1)	(0,2)	[-0.42;0.58]	[-0.6;0.56]	[-0.38;0.58]	[-0.6;0.48]	[-0.51;0.67]	[-0.55;0.59]
		(0,2)	(0,2)	[-0.75;0.69]	[-0.69;0.87]	[-0.66;0.69]	[-0.56;0.69]	[-0.47;0.69]	[-0.68;0.63]
	1	(-0.5,1)	(0.5,1)	[-1.09;1.3]	[-0.82;0.65]	[-0.26;1.31]	[-0.93;0.14]	[0.8;2.2]	[-1.63;-0.54]
		(-0.5,1)	(0.5,2)	[-0.36;0.85]	[-0.63;0.57]	[-0.18;0.97]	[-0.72;0.28]	[0.51;2.14]	[-1.13;-0.1]
		(-0.5,2)	(0.5,2)	[-0.96;1.07]	[-0.69;0.69]	[-0.68;1]	[-0.77;0.47]	[0;1.32]	[-0.98;0.04]
	2	(-1,1)	(1,1)	[-1.01;1.45]	[-0.63;0.51]	[0.26;2.16]	[-1.05;-0.18]	[2.66;4.95]	[-2.7;-1.61]
		(-1,1)	(1,2)	[-0.54;1.29]	[-0.49;0.68]	[0.02;1.77]	[-0.79;0.22]	[1.88;4.48]	[-1.71;-0.54]
		(-1,2)	(1,2)	[-0.91;1.39]	[-0.64;0.58]	[-0.58;1.41]	[-0.75;0.32]	[0.41;2.61]	[-1.45;-0.43]

IP: Item Parameter; PP: Person Parameter

Table 7: Relative Bias when $I = 20$ and $N = 1000$

**Simulation Input**				**Precision: Relative Bias Quantiles [Q1;Q3]**					
Cor.	Shift	IP (μ_δ, σ_δ)		PP $\sigma_\theta = 0.5$		PP $\sigma_\theta = 1$		PP $\sigma_\theta = 2$	
ρ_{sim}	Δ_{XY}	Scale X	Scale Y	X to Y	Y to X	X to Y	Y to X	X to Y	Y to X
0.25	0	(0,1)	(0,1)	[-0.36;0.29]	[-0.3;0.35]	[-0.55;0.45]	[-0.44;0.47]	[-1.08;0.9]	[-0.99;1.04]
		(0,1)	(0,2)	[-0.14;0.17]	[-0.25;0.23]	[-0.35;0.34]	[-0.39;0.37]	[-1.02;0.91]	[-0.9;1.01]
		(0,2)	(0,2)	[-0.28;0.23]	[-0.24;0.3]	[-0.47;0.37]	[-0.45;0.42]	[-1.15;0.98]	[-1.13;1.01]
	1	(-0.5,1)	(0.5,1)	[-1.11;-0.04]	[0.05;0.75]	[-2.56;-1.39]	[1.02;1.72]	[-5.61;-3.45]	[2.71;3.97]
		(-0.5,1)	(0.5,2)	[-0.53;-0.02]	[0.11;0.59]	[-1.71;-0.64]	[0.64;1.37]	[-4.74;-2.04]	[1.59;3.13]
		(-0.5,2)	(0.5,2)	[-0.7;0.16]	[-0.11;0.45]	[-1.52;-0.23]	[0.18;1.03]	[-3.68;-1.11]	[0.94;2.64]
	2	(-1,1)	(1,1)	[-1.92;-0.77]	[0.37;0.85]	[-5.21;-3.69]	[1.82;2.44]	[-11.25;-8.38]	[5.03;6.01]
		(-1,1)	(1,2)	[-1.08;-0.29]	[0.34;0.83]	[-3.38;-1.88]	[1.26;1.92]	[-8.94;-5.57]	[3.32;4.58]
		(-1,2)	(1,2)	[-0.96;0.01]	[-0.04;0.54]	[-2.6;-1.07]	[0.72;1.37]	[-6.71;-3.55]	[2.36;3.74]
0.5	0	(0,1)	(0,1)	[-0.38;0.34]	[-0.33;0.39]	[-0.36;0.31]	[-0.35;0.37]	[-0.73;0.61]	[-0.67;0.71]
		(0,1)	(0,2)	[-0.14;0.14]	[-0.2;0.18]	[-0.24;0.22]	[-0.33;0.28]	[-0.69;0.58]	[-0.59;0.67]
		(0,2)	(0,2)	[-0.26;0.26]	[-0.22;0.29]	[-0.37;0.31]	[-0.32;0.33]	[-0.81;0.71]	[-0.78;0.68]
	1	(-0.5,1)	(0.5,1)	[-0.78;0.17]	[-0.08;0.55]	[-1.68;-0.78]	[0.56;1.17]	[-3.68;-2.19]	[1.75;2.65]
		(-0.5,1)	(0.5,2)	[-0.36;0.07]	[0.04;0.45]	[-1.18;-0.38]	[0.41;0.9]	[-3.03;-1.33]	[1.05;2.07]
		(-0.5,2)	(0.5,2)	[-0.51;0.23]	[-0.16;0.4]	[-1.01;-0.09]	[0.06;0.72]	[-2.39;-0.69]	[0.63;1.68]
	2	(-1,1)	(1,1)	[-1.47;-0.35]	[0.15;0.64]	[-3.35;-2.19]	[1.11;1.59]	[-7.3;-5.3]	[3.18;3.89]
		(-1,1)	(1,2)	[-0.77;-0.06]	[0.19;0.71]	[-2.17;-1.13]	[0.78;1.3]	[-5.57;-3.41]	[2.14;2.99]
		(-1,2)	(1,2)	[-0.76;0.16]	[-0.1;0.44]	[-1.71;-0.67]	[0.37;0.93]	[-4.15;-2.27]	[1.56;2.34]
0.75	0	(0,1)	(0,1)	[-0.37;0.37]	[-0.34;0.38]	[-0.29;0.3]	[-0.3;0.29]	[-0.38;0.34]	[-0.36;0.39]
		(0,1)	(0,2)	[-0.13;0.13]	[-0.21;0.18]	[-0.17;0.13]	[-0.19;0.18]	[-0.3;0.28]	[-0.27;0.33]
		(0,2)	(0,2)	[-0.28;0.23]	[-0.21;0.32]	[-0.24;0.23]	[-0.24;0.25]	[-0.32;0.36]	[-0.37;0.34]
	1	(-0.5,1)	(0.5,1)	[-0.62;0.34]	[-0.24;0.39]	[-0.77;-0.14]	[0.12;0.54]	[-1.62;-0.86]	[0.66;1.17]
		(-0.5,1)	(0.5,2)	[-0.22;0.19]	[-0.03;0.35]	[-0.58;-0.05]	[0.11;0.51]	[-1.28;-0.51]	[0.46;0.94]
		(-0.5,2)	(0.5,2)	[-0.42;0.33]	[-0.18;0.31]	[-0.53;0.11]	[-0.03;0.41]	[-1.11;-0.24]	[0.21;0.81]
	2	(-1,1)	(1,1)	[-0.92;0.2]	[-0.07;0.41]	[-1.57;-0.75]	[0.36;0.74]	[-2.89;-1.89]	[1.13;1.59]
		(-1,1)	(1,2)	[-0.49;0.22]	[0.02;0.5]	[-1.01;-0.38]	[0.27;0.69]	[-2.09;-1.13]	[0.85;1.27]
		(-1,2)	(1,2)	[-0.61;0.38]	[-0.21;0.28]	[-0.92;-0.1]	[0.08;0.52]	[-1.88;-0.88]	[0.58;1.06]
1	0	(0,1)	(0,1)	[-0.39;0.38]	[-0.38;0.35]	[-0.3;0.37]	[-0.33;0.33]	[-0.27;0.3]	[-0.28;0.3]
		(0,1)	(0,2)	[-0.12;0.18]	[-0.16;0.19]	[-0.13;0.16]	[-0.18;0.2]	[-0.19;0.24]	[-0.22;0.18]
		(0,2)	(0,2)	[-0.29;0.27]	[-0.24;0.29]	[-0.28;0.23]	[-0.2;0.27]	[-0.24;0.24]	[-0.23;0.26]
	1	(-0.5,1)	(0.5,1)	[-0.39;0.56]	[-0.36;0.26]	[-0.08;0.57]	[-0.37;0.01]	[0.45;0.97]	[-0.7;-0.37]
		(-0.5,1)	(0.5,2)	[-0.13;0.29]	[-0.13;0.28]	[-0.09;0.39]	[-0.22;0.13]	[0.28;0.84]	[-0.45;-0.1]
		(-0.5,2)	(0.5,2)	[-0.32;0.39]	[-0.27;0.25]	[-0.25;0.39]	[-0.25;0.18]	[0.04;0.53]	[-0.37;0]
	2	(-1,1)	(1,1)	[-0.35;0.75]	[-0.3;0.17]	[0.2;0.96]	[-0.41;-0.07]	[1.4;2.31]	[-1.26;-0.81]
		(-1,1)	(1,2)	[-0.15;0.58]	[-0.15;0.28]	[0.02;0.62]	[-0.28;0.09]	[0.93;1.87]	[-0.72;-0.33]
		(-1,2)	(1,2)	[-0.38;0.6]	[-0.27;0.2]	[-0.22;0.56]	[-0.3;0.12]	[0.31;0.96]	[-0.56;-0.19]

IP: Item Parameter; PP: Person Parameter

Table 8: Score Transformation Precision when $I = 10$ and $N = 500$

**Simulation Input**				**Precision: NRMSE Quantiles [Q1;Q3]**					
Cor.	Shift	IP (μ_δ, σ_δ)		PP $\sigma_\theta = 0.5$		PP $\sigma_\theta = 1$		PP $\sigma_\theta = 2$	
ρ_{sim}	Δ_{XY}	Scale X	Scale Y	X to Y	Y to X	X to Y	Y to X	X to Y	Y to X
0.25	0	(0,1)	(0,1)	[0.18;0.19]	[0.18;0.2]	[0.25;0.27]	[0.25;0.27]	[0.37;0.39]	[0.37;0.39]
		(0,1)	(0,2)	[0.13;0.16]	[0.19;0.2]	[0.19;0.23]	[0.25;0.28]	[0.31;0.34]	[0.36;0.38]
		(0,2)	(0,2)	[0.14;0.17]	[0.14;0.17]	[0.2;0.23]	[0.2;0.23]	[0.3;0.34]	[0.3;0.34]
	1	(-0.5,1)	(0.5,1)	[0.17;0.19]	[0.18;0.19]	[0.25;0.27]	[0.25;0.27]	[0.36;0.38]	[0.36;0.38]
		(-0.5,1)	(0.5,2)	[0.13;0.16]	[0.18;0.2]	[0.19;0.22]	[0.25;0.27]	[0.3;0.34]	[0.36;0.38]
		(-0.5,2)	(0.5,2)	[0.14;0.16]	[0.14;0.17]	[0.19;0.23]	[0.19;0.23]	[0.3;0.34]	[0.3;0.34]
	2	(-1,1)	(1,1)	[0.17;0.18]	[0.17;0.19]	[0.23;0.26]	[0.23;0.26]	[0.35;0.38]	[0.35;0.38]
		(-1,1)	(1,2)	[0.13;0.16]	[0.17;0.19]	[0.19;0.22]	[0.23;0.26]	[0.29;0.33]	[0.35;0.37]
		(-1,2)	(1,2)	[0.13;0.16]	[0.14;0.16]	[0.18;0.22]	[0.19;0.22]	[0.29;0.33]	[0.29;0.33]
0.5	0	(0,1)	(0,1)	[0.17;0.18]	[0.17;0.18]	[0.22;0.24]	[0.22;0.24]	[0.31;0.33]	[0.31;0.33]
		(0,1)	(0,2)	[0.13;0.15]	[0.17;0.19]	[0.17;0.2]	[0.22;0.24]	[0.26;0.29]	[0.31;0.33]
		(0,2)	(0,2)	[0.13;0.16]	[0.13;0.16]	[0.17;0.2]	[0.17;0.2]	[0.26;0.29]	[0.26;0.29]
	1	(-0.5,1)	(0.5,1)	[0.16;0.18]	[0.16;0.18]	[0.21;0.23]	[0.22;0.23]	[0.31;0.32]	[0.31;0.33]
		(-0.5,1)	(0.5,2)	[0.12;0.15]	[0.17;0.19]	[0.17;0.2]	[0.22;0.24]	[0.25;0.29]	[0.3;0.33]
		(-0.5,2)	(0.5,2)	[0.13;0.15]	[0.13;0.16]	[0.17;0.2]	[0.17;0.2]	[0.25;0.29]	[0.25;0.29]
	2	(-1,1)	(1,1)	[0.15;0.17]	[0.15;0.17]	[0.21;0.23]	[0.21;0.23]	[0.3;0.32]	[0.3;0.32]
		(-1,1)	(1,2)	[0.12;0.15]	[0.16;0.18]	[0.16;0.19]	[0.21;0.23]	[0.25;0.28]	[0.3;0.32]
		(-1,2)	(1,2)	[0.13;0.15]	[0.13;0.15]	[0.16;0.19]	[0.17;0.2]	[0.25;0.28]	[0.25;0.28]
0.75	0	(0,1)	(0,1)	[0.15;0.16]	[0.15;0.17]	[0.18;0.19]	[0.18;0.2]	[0.24;0.25]	[0.24;0.25]
		(0,1)	(0,2)	[0.12;0.14]	[0.16;0.18]	[0.14;0.16]	[0.19;0.2]	[0.2;0.22]	[0.24;0.25]
		(0,2)	(0,2)	[0.12;0.15]	[0.12;0.15]	[0.15;0.17]	[0.15;0.17]	[0.2;0.22]	[0.2;0.22]
	1	(-0.5,1)	(0.5,1)	[0.15;0.16]	[0.15;0.16]	[0.18;0.19]	[0.18;0.19]	[0.23;0.25]	[0.23;0.25]
		(-0.5,1)	(0.5,2)	[0.12;0.14]	[0.16;0.17]	[0.14;0.16]	[0.18;0.2]	[0.19;0.22]	[0.24;0.25]
		(-0.5,2)	(0.5,2)	[0.12;0.14]	[0.12;0.15]	[0.14;0.17]	[0.15;0.17]	[0.2;0.22]	[0.19;0.22]
	2	(-1,1)	(1,1)	[0.14;0.16]	[0.14;0.16]	[0.17;0.19]	[0.17;0.19]	[0.23;0.25]	[0.23;0.25]
		(-1,1)	(1,2)	[0.11;0.14]	[0.15;0.17]	[0.14;0.16]	[0.18;0.19]	[0.19;0.21]	[0.23;0.25]
		(-1,2)	(1,2)	[0.12;0.14]	[0.12;0.14]	[0.14;0.16]	[0.14;0.17]	[0.19;0.22]	[0.19;0.22]
1	0	(0,1)	(0,1)	[0.14;0.15]	[0.14;0.15]	[0.13;0.14]	[0.13;0.14]	[0.12;0.12]	[0.12;0.12]
		(0,1)	(0,2)	[0.11;0.12]	[0.15;0.16]	[0.11;0.12]	[0.14;0.15]	[0.1;0.11]	[0.12;0.13]
		(0,2)	(0,2)	[0.11;0.13]	[0.11;0.13]	[0.11;0.13]	[0.11;0.13]	[0.11;0.12]	[0.11;0.12]
	1	(-0.5,1)	(0.5,1)	[0.14;0.15]	[0.13;0.15]	[0.13;0.14]	[0.13;0.14]	[0.12;0.13]	[0.12;0.13]
		(-0.5,1)	(0.5,2)	[0.11;0.12]	[0.14;0.16]	[0.11;0.12]	[0.14;0.15]	[0.1;0.11]	[0.12;0.13]
		(-0.5,2)	(0.5,2)	[0.11;0.13]	[0.11;0.13]	[0.11;0.13]	[0.11;0.13]	[0.11;0.12]	[0.11;0.12]
	2	(-1,1)	(1,1)	[0.13;0.15]	[0.13;0.15]	[0.13;0.14]	[0.13;0.14]	[0.12;0.14]	[0.12;0.14]
		(-1,1)	(1,2)	[0.1;0.12]	[0.14;0.16]	[0.11;0.12]	[0.13;0.15]	[0.11;0.12]	[0.12;0.14]
		(-1,2)	(1,2)	[0.11;0.13]	[0.11;0.13]	[0.11;0.13]	[0.11;0.13]	[0.11;0.12]	[0.11;0.12]

IP: Item Parameter; PP: Person Parameter; NRMSE: Normalized Root Mean Squared Error

Table 9: Score Transformation Precision when $I = 20$ and $N = 500$

**Simulation Input**				**Precision: NRMSE Quantiles [Q1;Q3]**					
Cor.	Shift	IP (μ_δ, σ_δ)		PP $\sigma_\theta = 0.5$		PP $\sigma_\theta = 1$		PP $\sigma_\theta = 2$	
ρ_{sim}	Δ_{XY}	Scale X	Scale Y	X to Y	Y to X	X to Y	Y to X	X to Y	Y to X
0.25	0	(0,1)	(0,1)	[0.15;0.16]	[0.15;0.16]	[0.23;0.25]	[0.24;0.25]	[0.36;0.38]	[0.36;0.37]
		(0,1)	(0,2)	[0.11;0.13]	[0.16;0.17]	[0.18;0.2]	[0.24;0.25]	[0.3;0.33]	[0.35;0.37]
		(0,2)	(0,2)	[0.12;0.13]	[0.12;0.14]	[0.18;0.2]	[0.18;0.21]	[0.29;0.32]	[0.29;0.32]
	1	(-0.5,1)	(0.5,1)	[0.15;0.16]	[0.15;0.16]	[0.23;0.25]	[0.23;0.25]	[0.35;0.37]	[0.36;0.37]
		(-0.5,1)	(0.5,2)	[0.11;0.13]	[0.15;0.16]	[0.18;0.2]	[0.23;0.25]	[0.3;0.33]	[0.35;0.37]
		(-0.5,2)	(0.5,2)	[0.12;0.13]	[0.12;0.13]	[0.18;0.2]	[0.18;0.2]	[0.29;0.32]	[0.29;0.32]
	2	(-1,1)	(1,1)	[0.14;0.15]	[0.14;0.15]	[0.22;0.24]	[0.22;0.24]	[0.35;0.37]	[0.35;0.37]
		(-1,1)	(1,2)	[0.11;0.13]	[0.14;0.15]	[0.17;0.2]	[0.22;0.23]	[0.29;0.32]	[0.34;0.36]
		(-1,2)	(1,2)	[0.11;0.13]	[0.11;0.13]	[0.17;0.2]	[0.17;0.2]	[0.29;0.32]	[0.29;0.32]
0.5	0	(0,1)	(0,1)	[0.14;0.15]	[0.14;0.15]	[0.2;0.21]	[0.2;0.21]	[0.3;0.31]	[0.3;0.31]
		(0,1)	(0,2)	[0.1;0.12]	[0.14;0.15]	[0.15;0.17]	[0.2;0.21]	[0.25;0.27]	[0.3;0.31]
		(0,2)	(0,2)	[0.11;0.12]	[0.11;0.12]	[0.15;0.17]	[0.15;0.18]	[0.25;0.27]	[0.24;0.27]
	1	(-0.5,1)	(0.5,1)	[0.13;0.14]	[0.13;0.14]	[0.2;0.21]	[0.2;0.21]	[0.29;0.31]	[0.3;0.31]
		(-0.5,1)	(0.5,2)	[0.1;0.12]	[0.14;0.15]	[0.15;0.17]	[0.2;0.21]	[0.25;0.27]	[0.29;0.31]
		(-0.5,2)	(0.5,2)	[0.11;0.12]	[0.11;0.12]	[0.15;0.17]	[0.15;0.17]	[0.24;0.27]	[0.24;0.27]
	2	(-1,1)	(1,1)	[0.12;0.14]	[0.13;0.14]	[0.19;0.2]	[0.19;0.2]	[0.29;0.31]	[0.29;0.31]
		(-1,1)	(1,2)	[0.1;0.11]	[0.13;0.14]	[0.15;0.17]	[0.19;0.2]	[0.24;0.27]	[0.29;0.3]
		(-1,2)	(1,2)	[0.1;0.12]	[0.1;0.12]	[0.15;0.17]	[0.15;0.17]	[0.24;0.26]	[0.24;0.26]
0.75	0	(0,1)	(0,1)	[0.12;0.13]	[0.12;0.13]	[0.16;0.17]	[0.16;0.17]	[0.22;0.23]	[0.22;0.23]
		(0,1)	(0,2)	[0.09;0.1]	[0.12;0.13]	[0.12;0.14]	[0.16;0.17]	[0.18;0.2]	[0.22;0.23]
		(0,2)	(0,2)	[0.1;0.11]	[0.1;0.11]	[0.12;0.14]	[0.12;0.14]	[0.18;0.2]	[0.18;0.2]
	1	(-0.5,1)	(0.5,1)	[0.12;0.12]	[0.12;0.12]	[0.15;0.16]	[0.15;0.16]	[0.22;0.23]	[0.22;0.23]
		(-0.5,1)	(0.5,2)	[0.09;0.1]	[0.12;0.13]	[0.12;0.14]	[0.16;0.17]	[0.18;0.2]	[0.22;0.23]
		(-0.5,2)	(0.5,2)	[0.09;0.11]	[0.09;0.11]	[0.12;0.14]	[0.12;0.14]	[0.18;0.2]	[0.18;0.2]
	2	(-1,1)	(1,1)	[0.11;0.12]	[0.11;0.12]	[0.15;0.16]	[0.15;0.16]	[0.21;0.23]	[0.22;0.23]
		(-1,1)	(1,2)	[0.09;0.1]	[0.11;0.12]	[0.12;0.13]	[0.15;0.16]	[0.18;0.2]	[0.21;0.23]
		(-1,2)	(1,2)	[0.09;0.1]	[0.09;0.1]	[0.12;0.13]	[0.12;0.14]	[0.18;0.2]	[0.18;0.2]
1	0	(0,1)	(0,1)	[0.1;0.1]	[0.1;0.1]	[0.09;0.1]	[0.09;0.1]	[0.08;0.09]	[0.08;0.09]
		(0,1)	(0,2)	[0.08;0.09]	[0.1;0.11]	[0.08;0.08]	[0.1;0.11]	[0.07;0.08]	[0.09;0.09]
		(0,2)	(0,2)	[0.08;0.09]	[0.08;0.09]	[0.08;0.09]	[0.08;0.09]	[0.08;0.08]	[0.07;0.08]
	1	(-0.5,1)	(0.5,1)	[0.1;0.1]	[0.1;0.1]	[0.09;0.1]	[0.09;0.1]	[0.08;0.09]	[0.08;0.09]
		(-0.5,1)	(0.5,2)	[0.08;0.09]	[0.1;0.11]	[0.08;0.08]	[0.1;0.11]	[0.07;0.08]	[0.09;0.09]
		(-0.5,2)	(0.5,2)	[0.08;0.09]	[0.08;0.09]	[0.08;0.09]	[0.08;0.09]	[0.08;0.08]	[0.08;0.08]
	2	(-1,1)	(1,1)	[0.09;0.1]	[0.09;0.1]	[0.09;0.1]	[0.09;0.1]	[0.09;0.1]	[0.09;0.1]
		(-1,1)	(1,2)	[0.08;0.09]	[0.1;0.11]	[0.08;0.09]	[0.1;0.1]	[0.08;0.09]	[0.09;0.1]
		(-1,2)	(1,2)	[0.08;0.09]	[0.08;0.09]	[0.08;0.09]	[0.08;0.09]	[0.08;0.09]	[0.08;0.08]

IP: Item Parameter; PP: Person Parameter; NRMSE: Normalized Root Mean Squared Error

Table 10: Score Transformation Precision when $I = 10$ and $N = 1000$

**Simulation Input**				**Precision: NRMSE Quantiles [Q1;Q3]**					
Cor.	Shift	IP (μ_δ, σ_δ)		PP $\sigma_\theta = 0.5$		PP $\sigma_\theta = 1$		PP $\sigma_\theta = 2$	
ρ_{sim}	Δ_{XY}	Scale X	Scale Y	X to Y	Y to X	X to Y	Y to X	X to Y	Y to X
0.25	0	(0,1)	(0,1)	[0.18;0.19]	[0.18;0.19]	[0.25;0.27]	[0.25;0.27]	[0.37;0.39]	[0.37;0.39]
		(0,1)	(0,2)	[0.14;0.16]	[0.19;0.2]	[0.19;0.23]	[0.26;0.27]	[0.31;0.34]	[0.37;0.38]
		(0,2)	(0,2)	[0.14;0.17]	[0.14;0.17]	[0.2;0.23]	[0.2;0.23]	[0.3;0.34]	[0.3;0.34]
	1	(-0.5,1)	(0.5,1)	[0.18;0.19]	[0.18;0.19]	[0.25;0.27]	[0.25;0.27]	[0.36;0.38]	[0.37;0.38]
		(-0.5,1)	(0.5,2)	[0.13;0.16]	[0.18;0.2]	[0.19;0.22]	[0.25;0.27]	[0.3;0.34]	[0.36;0.38]
		(-0.5,2)	(0.5,2)	[0.14;0.17]	[0.14;0.17]	[0.19;0.23]	[0.2;0.23]	[0.3;0.34]	[0.3;0.34]
	2	(-1,1)	(1,1)	[0.17;0.18]	[0.17;0.19]	[0.23;0.26]	[0.24;0.26]	[0.35;0.37]	[0.35;0.37]
		(-1,1)	(1,2)	[0.13;0.16]	[0.17;0.19]	[0.18;0.22]	[0.24;0.26]	[0.29;0.33]	[0.35;0.37]
		(-1,2)	(1,2)	[0.13;0.16]	[0.14;0.16]	[0.19;0.22]	[0.19;0.22]	[0.29;0.33]	[0.3;0.33]
0.5	0	(0,1)	(0,1)	[0.17;0.18]	[0.17;0.18]	[0.22;0.24]	[0.22;0.24]	[0.31;0.33]	[0.31;0.33]
		(0,1)	(0,2)	[0.13;0.15]	[0.18;0.19]	[0.17;0.2]	[0.23;0.24]	[0.26;0.29]	[0.31;0.33]
		(0,2)	(0,2)	[0.13;0.16]	[0.13;0.16]	[0.17;0.2]	[0.17;0.2]	[0.26;0.29]	[0.26;0.29]
	1	(-0.5,1)	(0.5,1)	[0.16;0.18]	[0.16;0.18]	[0.22;0.23]	[0.22;0.23]	[0.31;0.32]	[0.31;0.32]
		(-0.5,1)	(0.5,2)	[0.13;0.15]	[0.17;0.19]	[0.17;0.2]	[0.22;0.24]	[0.25;0.29]	[0.31;0.32]
		(-0.5,2)	(0.5,2)	[0.13;0.16]	[0.13;0.16]	[0.17;0.2]	[0.17;0.2]	[0.25;0.29]	[0.25;0.29]
	2	(-1,1)	(1,1)	[0.15;0.17]	[0.16;0.17]	[0.21;0.23]	[0.21;0.23]	[0.3;0.32]	[0.3;0.32]
		(-1,1)	(1,2)	[0.12;0.15]	[0.16;0.18]	[0.16;0.19]	[0.21;0.23]	[0.25;0.28]	[0.3;0.32]
		(-1,2)	(1,2)	[0.13;0.15]	[0.13;0.15]	[0.16;0.19]	[0.17;0.2]	[0.25;0.28]	[0.25;0.28]
0.75	0	(0,1)	(0,1)	[0.15;0.16]	[0.15;0.16]	[0.18;0.19]	[0.18;0.19]	[0.24;0.25]	[0.24;0.25]
		(0,1)	(0,2)	[0.12;0.14]	[0.16;0.18]	[0.14;0.16]	[0.19;0.2]	[0.2;0.22]	[0.24;0.25]
		(0,2)	(0,2)	[0.12;0.15]	[0.13;0.15]	[0.15;0.17]	[0.15;0.17]	[0.2;0.22]	[0.2;0.22]
	1	(-0.5,1)	(0.5,1)	[0.15;0.16]	[0.15;0.16]	[0.18;0.19]	[0.18;0.19]	[0.24;0.25]	[0.24;0.25]
		(-0.5,1)	(0.5,2)	[0.12;0.14]	[0.16;0.17]	[0.14;0.16]	[0.18;0.2]	[0.19;0.22]	[0.24;0.25]
		(-0.5,2)	(0.5,2)	[0.12;0.14]	[0.12;0.15]	[0.15;0.17]	[0.15;0.17]	[0.2;0.22]	[0.2;0.22]
	2	(-1,1)	(1,1)	[0.14;0.16]	[0.14;0.16]	[0.17;0.19]	[0.17;0.19]	[0.23;0.25]	[0.23;0.25]
		(-1,1)	(1,2)	[0.11;0.14]	[0.15;0.17]	[0.14;0.16]	[0.18;0.19]	[0.19;0.21]	[0.23;0.25]
		(-1,2)	(1,2)	[0.12;0.14]	[0.12;0.14]	[0.14;0.16]	[0.14;0.17]	[0.19;0.22]	[0.19;0.22]
1	0	(0,1)	(0,1)	[0.14;0.15]	[0.14;0.15]	[0.13;0.14]	[0.13;0.14]	[0.12;0.12]	[0.12;0.12]
		(0,1)	(0,2)	[0.11;0.12]	[0.15;0.16]	[0.11;0.12]	[0.14;0.15]	[0.1;0.11]	[0.12;0.13]
		(0,2)	(0,2)	[0.11;0.13]	[0.11;0.13]	[0.11;0.13]	[0.11;0.13]	[0.11;0.12]	[0.11;0.12]
	1	(-0.5,1)	(0.5,1)	[0.14;0.15]	[0.14;0.15]	[0.13;0.14]	[0.13;0.14]	[0.12;0.13]	[0.12;0.13]
		(-0.5,1)	(0.5,2)	[0.11;0.12]	[0.14;0.16]	[0.11;0.12]	[0.14;0.15]	[0.1;0.11]	[0.12;0.13]
		(-0.5,2)	(0.5,2)	[0.11;0.13]	[0.11;0.13]	[0.11;0.13]	[0.11;0.13]	[0.11;0.12]	[0.11;0.12]
	2	(-1,1)	(1,1)	[0.13;0.14]	[0.13;0.15]	[0.13;0.14]	[0.13;0.14]	[0.12;0.14]	[0.13;0.14]
		(-1,1)	(1,2)	[0.11;0.12]	[0.14;0.16]	[0.11;0.12]	[0.14;0.15]	[0.11;0.12]	[0.13;0.14]
		(-1,2)	(1,2)	[0.11;0.13]	[0.11;0.13]	[0.11;0.13]	[0.11;0.13]	[0.11;0.12]	[0.11;0.12]

IP: Item Parameter; PP: Person Parameter; NRMSE: Normalized Root Mean Squared Error

Table 11: Score Transformation Precision: $I = 20$ and $N = 1000$

**Simulation Input**				**Precision: NRMSE Quantiles [Q1;Q3]**					
Cor.	Shift	IP (μ_δ, σ_δ)		PP $\sigma_\theta = 0.5$		PP $\sigma_\theta = 1$		PP $\sigma_\theta = 2$	
ρ_{sim}	Δ_{XY}	Scale X	Scale Y	X to Y	Y to X	X to Y	Y to X	X to Y	Y to X
0.25	0	(0,1)	(0,1)	[0.15;0.16]	[0.15;0.16]	[0.24;0.25]	[0.24;0.25]	[0.36;0.37]	[0.36;0.37]
		(0,1)	(0,2)	[0.11;0.13]	[0.16;0.17]	[0.18;0.2]	[0.24;0.25]	[0.3;0.33]	[0.36;0.37]
		(0,2)	(0,2)	[0.12;0.13]	[0.12;0.13]	[0.18;0.2]	[0.18;0.21]	[0.3;0.32]	[0.3;0.32]
	1	(-0.5,1)	(0.5,1)	[0.15;0.16]	[0.15;0.16]	[0.23;0.25]	[0.23;0.25]	[0.36;0.37]	[0.36;0.37]
		(-0.5,1)	(0.5,2)	[0.11;0.13]	[0.15;0.16]	[0.18;0.2]	[0.23;0.25]	[0.3;0.33]	[0.35;0.37]
		(-0.5,2)	(0.5,2)	[0.12;0.13]	[0.12;0.13]	[0.18;0.2]	[0.18;0.2]	[0.3;0.32]	[0.29;0.32]
	2	(-1,1)	(1,1)	[0.14;0.15]	[0.14;0.15]	[0.22;0.23]	[0.22;0.24]	[0.35;0.36]	[0.35;0.37]
		(-1,1)	(1,2)	[0.11;0.13]	[0.14;0.15]	[0.17;0.2]	[0.22;0.23]	[0.3;0.32]	[0.34;0.36]
		(-1,2)	(1,2)	[0.11;0.13]	[0.11;0.13]	[0.17;0.2]	[0.17;0.2]	[0.29;0.32]	[0.29;0.32]
0.5	0	(0,1)	(0,1)	[0.14;0.15]	[0.14;0.15]	[0.2;0.21]	[0.2;0.21]	[0.3;0.31]	[0.3;0.31]
		(0,1)	(0,2)	[0.1;0.12]	[0.14;0.15]	[0.16;0.17]	[0.2;0.21]	[0.25;0.27]	[0.3;0.31]
		(0,2)	(0,2)	[0.11;0.12]	[0.11;0.12]	[0.16;0.17]	[0.16;0.18]	[0.25;0.27]	[0.25;0.27]
	1	(-0.5,1)	(0.5,1)	[0.13;0.14]	[0.13;0.14]	[0.2;0.21]	[0.2;0.21]	[0.3;0.31]	[0.3;0.31]
		(-0.5,1)	(0.5,2)	[0.1;0.12]	[0.14;0.15]	[0.15;0.17]	[0.2;0.21]	[0.25;0.27]	[0.3;0.31]
		(-0.5,2)	(0.5,2)	[0.11;0.12]	[0.11;0.12]	[0.15;0.17]	[0.15;0.17]	[0.25;0.27]	[0.24;0.27]
	2	(-1,1)	(1,1)	[0.13;0.14]	[0.13;0.14]	[0.19;0.2]	[0.19;0.2]	[0.29;0.3]	[0.29;0.31]
		(-1,1)	(1,2)	[0.1;0.11]	[0.13;0.14]	[0.15;0.17]	[0.19;0.2]	[0.24;0.27]	[0.29;0.3]
		(-1,2)	(1,2)	[0.1;0.12]	[0.1;0.12]	[0.15;0.17]	[0.15;0.17]	[0.24;0.26]	[0.24;0.26]
0.75	0	(0,1)	(0,1)	[0.12;0.13]	[0.12;0.13]	[0.16;0.17]	[0.16;0.17]	[0.22;0.23]	[0.22;0.23]
		(0,1)	(0,2)	[0.09;0.1]	[0.12;0.13]	[0.12;0.14]	[0.16;0.17]	[0.18;0.2]	[0.22;0.23]
		(0,2)	(0,2)	[0.09;0.11]	[0.09;0.11]	[0.12;0.14]	[0.12;0.14]	[0.18;0.2]	[0.18;0.2]
	1	(-0.5,1)	(0.5,1)	[0.12;0.12]	[0.12;0.12]	[0.15;0.16]	[0.15;0.16]	[0.22;0.23]	[0.22;0.23]
		(-0.5,1)	(0.5,2)	[0.09;0.1]	[0.12;0.13]	[0.12;0.14]	[0.16;0.17]	[0.18;0.2]	[0.22;0.23]
		(-0.5,2)	(0.5,2)	[0.09;0.11]	[0.09;0.11]	[0.12;0.14]	[0.12;0.14]	[0.18;0.2]	[0.18;0.2]
	2	(-1,1)	(1,1)	[0.11;0.12]	[0.11;0.12]	[0.15;0.16]	[0.15;0.16]	[0.22;0.23]	[0.22;0.23]
		(-1,1)	(1,2)	[0.09;0.1]	[0.11;0.12]	[0.12;0.13]	[0.15;0.16]	[0.18;0.2]	[0.22;0.23]
		(-1,2)	(1,2)	[0.09;0.1]	[0.09;0.1]	[0.12;0.13]	[0.12;0.14]	[0.18;0.2]	[0.18;0.2]
1	0	(0,1)	(0,1)	[0.1;0.1]	[0.1;0.1]	[0.09;0.1]	[0.09;0.1]	[0.08;0.09]	[0.08;0.09]
		(0,1)	(0,2)	[0.08;0.09]	[0.1;0.11]	[0.08;0.08]	[0.1;0.11]	[0.07;0.08]	[0.09;0.09]
		(0,2)	(0,2)	[0.08;0.09]	[0.08;0.09]	[0.08;0.09]	[0.08;0.09]	[0.08;0.08]	[0.08;0.08]
	1	(-0.5,1)	(0.5,1)	[0.1;0.1]	[0.1;0.1]	[0.09;0.1]	[0.09;0.1]	[0.08;0.09]	[0.08;0.09]
		(-0.5,1)	(0.5,2)	[0.08;0.09]	[0.1;0.11]	[0.08;0.08]	[0.1;0.1]	[0.07;0.08]	[0.09;0.09]
		(-0.5,2)	(0.5,2)	[0.08;0.09]	[0.08;0.09]	[0.08;0.09]	[0.08;0.09]	[0.08;0.08]	[0.08;0.08]
	2	(-1,1)	(1,1)	[0.09;0.1]	[0.09;0.1]	[0.09;0.1]	[0.09;0.1]	[0.09;0.1]	[0.09;0.1]
		(-1,1)	(1,2)	[0.08;0.08]	[0.1;0.11]	[0.08;0.09]	[0.1;0.1]	[0.08;0.09]	[0.09;0.1]
		(-1,2)	(1,2)	[0.08;0.09]	[0.08;0.09]	[0.08;0.09]	[0.08;0.09]	[0.08;0.08]	[0.08;0.08]

IP: Item Parameter; PP: Person Parameter; NRMSE: Normalized Root Mean Squared Error
